# Acupoint temperature as a biomarker: infrared thermography in the diagnosis of adolescents with major depressive disorder

**DOI:** 10.3389/fpsyt.2026.1806676

**Published:** 2026-04-29

**Authors:** Siying Qu, Mingqi Tu, Junyan Jin, Xiaoting Wu, Sangsang Xiong, Nisang Chen, Zelin Yu, Jianqiao Fang, Xiaomei Shao

**Affiliations:** 1 The Third Clinical Medical College, The Third Affiliated Hospital of Zhejiang Chinese Medical University, Hangzhou, China; 2 Department of Acupuncture, The Affiliated Hangzhou First People’s Hospital, Hangzhou, China; 3 Department of Acupuncture, Shaoxing Hospital of Traditional Chinese Medicine, Shaoxing, China; 4 Department of Rehabilitation Medicine, The Second Affiliated Hospital of Zhejiang University, Hangzhou, China; 5 Department of Massage, The First Affiliated Hospital of Zhejiang Chinese Medical University, Hangzhou, China

**Keywords:** adolescents, diagnostic model, infrared relative temperature of acupoint, infrared thermography, major depressive disorder

## Abstract

**Background:**

The prevalence of adolescent major depressive disorder (MDD) is rising; however, diagnosis relies on subjective measures due to a lack of objective biomarkers. This study explored infrared thermography (IRT) as a non-invasive tool to quantify thermal radiation characteristics of acupoints in adolescents with MDD. The objective was to establish diagnostic models based on acupoint temperature-derived biomarkers.

**Methods:**

A prospective, multi-center observational study enrolled 108 participants (65 adolescents with MDD and 43 healthy controls [HCs]). We first examined correlations between acupoint temperatures and depression severity using Pearson analysis. Multiple linear and binary logistic regression models were developed to diagnose MDD and assess severity. The diagnostic model for MDD was visualized as a nomogram and validated using Receiver Operating Characteristic (ROC) curves, Hosmer-Lemeshow tests, calibration plots, and decision curve analysis (DCA). Internal validation was performed using the bootstrap method.

**Results:**

Among 27 acupoints analyzed, adolescents with MDD exhibited altered acupoint temperatures at Taiyang (EX-HN5), Quchi (LI11), Yanggu (SI5), and Waiqiu (GB36). Subsequent Pearson correlation analysis revealed negative correlations between the infrared relative temperatures of Taiyang (EX-HN5), Quchi (LI11), and Waiqiu (GB36) and depression severity (*P* = 0.001, *r* = -0.319; *P* = 0.022, *r* = -0.229; *P* = 0.001, *r* = -0.325) and a weak positive correlation between the infrared relative temperature of Yanggu (SI5) and depression severity (*P* = 0.043, *r* = 0.202). Building on these findings, two diagnostic models were developed: a linear regression model for depression severity of adolescents (Y = 52.25-9.52*T_EX-HN5_-13.07*T_GB36_) and a logistic regression model for adolescents with MDD diagnosis (P = e^x^/(1+e^x^), x = 0.22-1.14*T_EX-HN5_+0.45*T_SI5_-2.19*T_GB36_). The nomogram-based model demonstrated good calibration (Hosmer-Lemeshow *P* = 0.855), discrimination (AUC = 0.785, 95%CI: 0.693 - 0.876), and clinical utility. Internal validation using the bootstrap method produced a C-index of 0.752 (95% CI: 0.617 - 0.877), further confirming the model’s robustness.

**Conclusions:**

In conclusion, acupoint temperature-based models show promising efficacy for the objective and non-invasive diagnosis and severity quantification of adolescents with MDD, offering valuable tools for early clinical intervention. Future studies should validate these findings across diverse populations and integrate multi-modal biomarkers to enhance diagnostic precision.

**Clinical Trial Registration:**

ClinicalTrials.gov, identifier NCT06750640.

## 1 Introduction

Major Depressive Disorder (MDD) is a mental health condition marked by a depressed mood, lack of interest, and diminished pleasure, along with symptoms such as anxiety, cognitive deficits, psychomotor problems, and potential suicidal thoughts (1, 2). Factors such as internet addiction, and academic stress (3–5), have contributed to an increased incidence in recent years, with a trend toward a younger age of onset (6). It is estimated that approximately one out of five adolescents is affected by MDD globally (7). Therefore, early screening and early treatment of MDD in adolescents are particularly important.

However, the existing process for diagnosing MDD relies heavily on the experience of clinical psychiatrists, professional assessment tools, and patients’ subjective narratives (8–10). Additionally, some symptoms of MDD in adolescents are insidious, such as declines in academic performance, poor concentration, and social difficulties (11), which can lead to misdiagnosis and inappropriate treatment and may be confused with attention deficit hyperactivity disorder, bipolar disorder, and other diseases (12, 13). Therefore, developing new therapeutic approaches and diagnostic methods is urgently needed.

According to the meridian theory, acupoints are the reaction points of disease. Modern studies have confirmed that skin temperature at acupoints changes when the body is experiencing pathological conditions (14–16). This suggests that changes in acupoint temperature can objectively respond to the symptoms of a disease. Emerging evidence suggests potential acupoint temperature alterations in patients with MDD (17); however, definitive evidence remains elusive in the current literature. Furthermore, many studies have focused on the role of acupoints in MDD treatment (18, 19), while overlooking their potential role in disease diagnosis. To date, there have been no systematic studies of acupoint temperatures using objective testing methods.

Infrared thermography (IRT), a non-invasive, radiation-free medical imaging technique, which accurately detects temperature changes, was used to measure skin temperature (20–22). IRT uses a thermal imager to capture infrared radiation energy and convert it into a visible image. Through IRT, an infrared “map” of the body is generated and can be displayed as a color image with the help of computer processing. The advent of IRT has opened up new possibilities for the diagnosis and treatment of many diseases (23–25). Therefore, IRT was employed to detect acupoint temperatures in this study.

With the growing trend of interdisciplinary research, the development of clinical predictive models has emerged as a popular and novel approach in medicine (26, 27). The predictive model may be more accurate and informative for disease risk assessment (diagnostic models) and prognosis evaluation (prognostic models) (28), thereby contributing to the rational allocation of medical resources and improving outcomes. It has been reported that diagnostic models for adolescents with MDD based on quantitative electroencephalography have been developed (29). Some researchers have developed MDD diagnostic models using structural magnetic resonance imaging scans, in which the temporal pole had the highest weight (30). However, considering the higher cost associated with these approaches, some limits to their generalizability remain. In addition, to the best of our knowledge, no diagnostic models for adolescents with MDD based on acupoint temperature have been reported.

In this study, we first evaluated the reproducibility of acupoint temperature measurement by IRT. Next, we explored the altered temperature of MDD-related acupoints and developed a diagnostic model for depression severity in adolescents and a diagnostic model for adolescents with MDD based on acupoint temperature. Our research findings provide new ideas, methods, and visualization tools for early screening, auxiliary diagnosis, and condition assessment of MDD in adolescents.

## 2 Methods

### 2.1 Study design

This multi-center cross-sectional study employed IRT to characterize acupoint temperature alterations in adolescents with MDD, with subsequent development of diagnostic models for depression severity and MDD. To ensure measurement reproducibility, we conducted a preliminary reliability study prior to the main research, involving 27 adolescents with MDD, in which two investigators independently performed IRT measurements on the 27 selected acupoints at two distinct time sessions to assess both inter- and intra-investigator consistency. Reliability was rigorously evaluated using the intraclass correlation coefficient (ICC) and Bland-Altman analysis. The results demonstrated excellent reliability, with ICC values ranging from 0.809 to 0.999 and 92.6%–100% of data points falling within the 95% limits of agreement (**Supplementary Material 1**). A supplementary analytical phase was implemented to evaluate bilateral acupoint temperatures (**Supplementary Material 2**).

The present study received ethical approval from the Ethics Committee Board of the participating hospital (approval numbers: the Third Affiliated Hospital of Zhejiang Chinese Medical University: ZSLL-KY-2022-001-01-05, Hangzhou First People’s Hospital: 2022-184(K)-02, and Tongde Hospital of Zhejiang Province: KY-20220905-0142-01-05). All subjects provided assent to participate in this study, while parents or guardians provided informed consent. This trial is registered at ClinicalTrials.gov with the registration number NCT06750640.

### 2.2 Participants

From May 2022 to May 2024, adolescents with MDD were recruited from the Third Affiliated Hospital of Zhejiang Chinese Medical University, Hangzhou First People’s Hospital, and Tongde Hospital of Zhejiang Province and were diagnosed based on the International Classification of Diseases-10 (ICD-10) by psychiatrists with 20 years of clinical experience. The Self-rating Depression Scale (SDS) was completed by all subjects to assess depression severity. Only patients with an SDS score greater than 53 were included in the study. In addition, gender- and age-matched adolescent volunteers with SDS scores < 53 were recruited from the physical examination center of the Third Affiliated Hospital of Zhejiang Chinese Medical University. The volunteers were further interviewed by a psychiatrist to screen for the presence of depressive symptoms. The exclusion criteria for both groups included: (a) severe anxiety, schizophrenia, or other serious mental disorders; (b) presence of pigmentation, redness, infection, or scarring at the skin sites of detection; (c) severe systemic diseases and their complications, serious infections, and other major medical conditions; (d) pregnancy, lactation, or being within ± 2 days of menstruation or ovulation; (e) body temperature ≥ 37.3°C; and (f) failure to complete the IRT measurement.

### 2.3 Acquisition of IRT images

All IRT examinations were conducted in a standardized environment adhering to the Thermographic Imaging in Sports and Exercise Medicine (TISEM) checklist to ensure methodological transparency and reproducibility (31, 32). Measurements were performed in a soundproof, draft-free room maintained at 26 ± 1 °C and 40 - 60% relative humidity. Additionally, no other sources of infrared radiation or direct sunlight were present during measurement. To minimize physiological confounders, all subjects were instructed to avoid food consumption, strenuous physical activity, smoking, and exposure to excessive low or high temperatures in the 1 h prior to the testing session and to abstain from any vasoactive substances such as caffeine or alcohol in the preceding 24 h. Furthermore, the skin at the detection sites was ensured to be clean and free of any topical agents (e.g., cosmetics, creams, or lotions). Before testing, participants stayed in the dedicated room for 20 min with the examination site fully exposed to allow for skin temperature stabilization.

A thermograph (NEC InfRec R450, Avio Infrared Technologies Co., Ltd., Japan; thermal sensitivity: < 0.025 °C, spatial resolution: 480 × 360 pixels) was used to record thermal images. To ensure measurement accuracy and reliability, the camera was calibrated annually by the manufacturer according to the recommended procedures and standards. The emissivity (ϵ) was set to 0.98 for human skin. The infrared camera was fixed on a tripod 1 m away from the subject and positioned perpendicular (90° angle) to the target surface to ensure image stability and accuracy. Appropriate body position was selected depending on the anatomical region being examined. Using the automatic interval saving shooting mode, thermal images were captured at 10‐s intervals, with three images taken for each subject.

### 2.4 The detection acupoints and analytical method

Prior to this trial, we performed a data-mining analysis of the literature on acupuncture for MDD (33). Considering that the chest, abdomen, and back are more private areas, the inclusion of acupoints from these areas would not be conducive to the broader applicability of the diagnostic model based on acupoint temperature. Accordingly, and in combination with traditional Chinese medicine theory, 27 acupoints with a high frequency of use in acupuncture treatment of MDD were selected as the test acupoints in the present study. The localization of regions and specific acupoints is shown in **Table 1** and **Table 2**.

**Table 1:** Anatomical regions and their boundaries for IRT measurement..

Regions	Location
Facial region	The triangular region between Yintang (GV29) and Yangbai (GB4) (on both sides).
Medial upper limb region	The measurement area between the transverse striation on the palm side of the wrist and the elbow transverse striae.
Lateral upper limb region	The measurement area between the transverse striation on the dorsal forearm of the wrist and the extension line of the connecting line between Quchi (LI11) and Xiaohai (SI8).
Medial lower limb region	The measurement area between the extension line between the medial end of the patellar base and the lateral end of the transverse popliteal fossa, and the extension line between the tips of the medial malleoli and Taixi (SI3).
Lateral lower limb region	The measurement area between the extension line between the medial end of the patellar base and the lateral end of the transverse popliteal fossa, and the extension line between the tips of the lateral malleoli and Kunlun (BL60).

**Table 2:** Locations of the 27 acupoints selected for IRT measurement.

Acupoints		Location
Face	Yintang (GV29)	On the forehead, at the midpoint between the two medial ends of the eyebrows.
	Shuigou (GV26)	On the face, at the intersection of the upper and top thirds of the Renzhong ditch.
	Taiyang (EX-HN5)	In the temple region, in the depression approximately one fingerbreadth posterior to the midpoint between the lateral end of the eyebrow and the outer canthus.
Medial upper limb	Chize (LU5)	On the cubital crease, on the radial side of the tendon of m. biceps brachii.
	Yuji (LU10)	At the midpoint of the palmar side of the first metacarpal bone, at the junction of the red and white skin.
	Neiguan (PC6)	2 cun above the transverse crease of the wrist, on the line connecting Quze (PC3) and Daling (PC7), between the tendons of m. flexor carpi radialis.
	Shenmen (HT7)	At the ulnar end of the transverse crease of the wrist, in the depression on the radial side of the tendon of m. flexor carpi ulnaris.
	Tongli (HT4)	On the radial side of the tendon of m. flexor carpi ulnaris, 1.5 cun above the transverse crease of the wrist.
	Shaohai (HT3)	When the elbow is flexed, the point is at the midpoint of the line connecting the medial end of the transverse cubital crease and the medial epicondyle of the humerus.
Lateral upper limb	Quchi (LI11)	When elbow is flexed at an angle of 90°, the point is at the midpoint of the lateral end of the transverse cubital crease and the lateral epicondyle of the humerus.
	Hegu (LI4)	On the dorsum of the hand, between the first and second metacarpal bones, in the top of the second metacarpal bone on the radial side.
	Pianli (LI6)	With elbow flexed, on the line connecting Yangxi (LI5) and Quchi (LI11), 3 cun above the wrist crease.
	Yanggu (SI5)	On the ulnar aspect of the wrist, in the depression between the styloid process of the ulna and the triquetral bone.
	Zhizheng (SI7)	On the dorsal ulnar aspect of the forearm, 5 cun above the transverse crease of the wrist, on the line connecting Yanggu (SI5) and Xiaohai (SI8).
Medial lower limb	Sanyinjiao (SP6)	3 cun above the medial malleolus, on the posterior border of the medial aspect of tibia.
	Yinlingquan (SP9)	In the depression of the lower border of the medial condyle of the tibia.
	Taixi (KI3)	Posterior to the medial malleolus, in the depression between the tip of the medial malleolus and tendo calcaneus.
	Zhaohai (KI6)	In the depression below the tip of the medial malleolus.
Lateral lower limb	Zusanli (ST36)	3 cun below Dubi (ST35), on finger-breadth (top finger) from the anterior crest of tibia.
	Fenglong (ST40)	8 cun superior to the external malleolus, lateral to Tiaokou (ST38), two finger-breadth (top finger) from the anterior crest of the tibia.
	Jiexi (ST41)	In the depression at the midpoint of the transverse crease of the ankle between the tendons of m. extensor hallucis longus and digitorum longus.
	Yanglingquan (GB34)	In the depression anterior and inferior to the head of the fibula.
	Waiqiu (GB36)	7 cun above the tip of the external malleolus, on the anterior border of the fibula.
	Qiuxu (GB40)	Anterior and inferior to the external malleolus, in the depression on the lateral side of the tendon of m. extensor digitorum longus.
	Shenmai (BL62)	In the depression directly below the external malleolus.
	Taichong (LR3)	On the dorsum of the foot, in the depression proximal to the first metatarsal space.
	Xingjian (LR2)	On the dorsum of the foot, proximal to the margin of the web between the first and second toes.

The anatomical positioning of all selected acupoints is defined according to the standard guidelines of traditional acupuncture nomenclature.

Cun is a traditional Chinese unit of measurement used in acupuncture and meridian theory to locate acupoints relative to the patient’s body, not fixed absolute dimensions. A common method for measuring a cun is to take the width of the transverse crease at the end segment of one’s own thumb as 1 cun.

An infrared imaging system and the accompanying software (InfRec Analyzer NS9500) were used for data analysis. For acupoint measurements, the Region of Interest (ROI) was set up as a standard circular region with a diameter of 1 cm (approximately 61 pixels), in accordance with a previous study (34). The average temperature of the acupoint ROI across the three infrared images was calculated and defined as the absolute infrared temperature of the acupoint. Furthermore, the absolute infrared temperature of a region was defined as the mean surface temperature of the entire delineated area within the respective anatomical region, which was automatically computed by the thermography analysis software based on all pixels within the boundary. Similarly, the absolute regional temperature was derived by averaging the regional temperature across the three images.

To minimize environmental and metabolic confounders, the infrared relative temperature of each acupoint was calculated by subtracting regional temperatures from acupoint values. The following is the method of calculating the infrared relative temperature of an acupoint:

Relative infrared temperature of acupoint = absolute infrared temperature of acupoint - absolute infrared temperature of the region where the acupoint is located.

**Figure 1:**
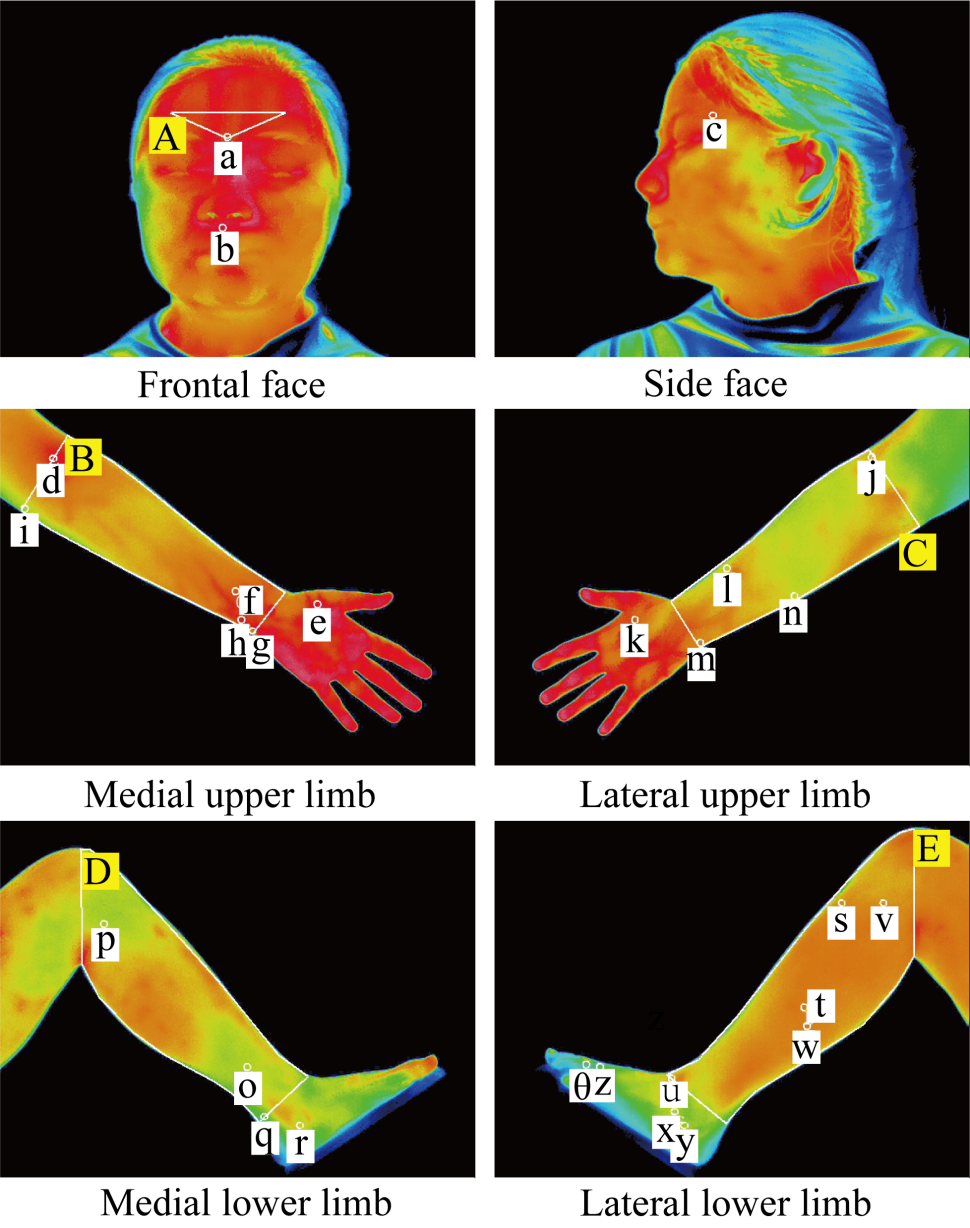
Locations of the 27 selected acupoints and anatomical regions for IRT measurement. These acupoints are represented by white hollow circles with a diameter of 1 cm and are indicated by lowercase letters or symbols with white backgrounds: a, Yintang (GV29); b, Shuigou (GV26); c, Taiyang (EX-HN5); d, Chize (LU5); e, Yuji (LU10); f, Neiguan (PC6); g, Shenmen (HT7); h, Tongli (HT4); i, Shaohai (HT3); j, Quchi (LI11); k, Hegu (LI4); l, Pianli (LI6); m, Yanggu (SI5); n, Zhizheng (SI7); o, Sanyinjiao (SP6); p, Yinlingquan (SP9); q, Taixi (KI3); r, Zhaohai (KI6); s, Zusanli (ST36); t, Fenglong (ST40); u, Jiexi (ST41); v, Yanglingquan (GB34); w, Waiqiu (GB36); x, Qiuxu (GB40); y, Shenmai (BL62); z, Taichong (LR3); θ, Xingjian (LR2). Five anatomical regions are delineated by solid white lines and indicated by uppercase letters with yellow backgrounds: **(A)** Facial region; **(B)** Medial upper limb region; **(C)** Lateral upper limb region; **(D)** Medial lower limb region; **(E)** Lateral lower limb region. Detailed definitions of the anatomical boundaries and acupoint locations are provided in **Tables 1** and **2**, respectively..

The detailed locations of acupoints and regional divisions are shown in **Figure 1**.

### 2.5 Statistical analysis

SPSS 25.0 (IBM SPSS Statistics for Windows, USA) and GraphPad Prism 8.0.2 (GraphPad Prism, USA) and R 4.2.3 (University of Auckland, New Zealand) were used for statistical analysis and graphing. All data from the MDD and HC groups were analyzed for outliers. Outliers, defined as values lying more than three standard deviations (SD) from the mean, were replaced by the group mean. All normally distributed data are presented as mean ± SD, and non-normally distributed data are presented as M (Q_1_, Q_3_). For data conforming to the normal distribution, the independent sample t-test was used for comparisons between groups; otherwise, the Mann-Whitney U test was applied. Count data were expressed as the number of cases (%), and a χ^2^ test was used for comparisons between the groups. A p-value of 0.05 was considered to indicate a statistically significant difference. Differential acupoints were filtered from comparisons between groups. Depression severity was rated using SDS (35). The severity of depression was analyzed by Pearson correlation analysis. Variables found to be statistically significantly associated with depression severity in the Pearson correlation analysis were included in the multiple linear regression analysis. A diagnostic model for depression severity in adolescents was then constructed, and the validity of the model was evaluated. Before conducting a binary logistic regression test, the study performed a colinearity test to ensure no multicollinearity symptoms existed between the independent variables. Binary logistic regression analyses were employed to construct the model for adolescent MDD diagnosis. Then, the regplot package in R was utilized to plot nomograms for visualization of the diagnostic model. Additionally, the Receiver Operating Characteristic (ROC) curves were drawn, and the area under the Receiver Operating Characteristic curve (AUC), specificity, and sensitivity were computed. Model fitness was assessed using the Hosmer-Lemeshow goodness-of-fit test. Calibration curves were utilized to assess the calibration ability of the model, while Decision curve analysis (DCA) was used to compare clinical net benefits. Bootstrap resampling with 1,000 iterations was used for internal validation to assess the model’s stability.

## 3 Result

### 3.1 Baseline characteristics

Initially, 43 HCs and 65 adolescents with MDD were enrolled. However, three adolescents from each group did not complete the examination, and two patients were re-diagnosed with bipolar disorder after the trial. Consequently, data from 100 participants were included in the final analysis. Baseline characteristics, including gender, age, and body mass index (BMI), were comparable between the MDD and HC groups, suggesting that the baseline conditions of the two groups were comparable (**Table 3**). Statistical significance was defined as *P* < 0.05 for all analyses.

**Table 3:** Baseline sociodemographic characteristics and anthropometric measurements of adolescents with MDD vs. HCs [n (%), M (Q1, Q3)].

Variables	HC group (n=40)	MDD group (n=60)	χ²/*z*	*P* value
Sex			0.20	0.656
Female	27 (67.50)	43 (71.67)		
Male	13 (32.50)	17 (28.33)		
Age (years)	15.00 (14.00, 16.00)	15.00 (14.00, 16.00)	-0.03	0.974
BMI (kg/m^2^)	20.42 (19.26, 21.61)	19.94 (18.73, 22.82)	-0.51	0.610

Data are presented as n (%) for categorical variables and median (Q1, Q3) for continuous variables. Group comparisons were performed using the χ² test for categorical variables and the Mann-Whitney U test for continuous variables. BMI, body mass index.

### 3.2 Inter−group comparison of the MDD and HC groups

Significant differences in infrared relative temperatures were observed between the MDD and HC groups. Adolescents with MDD exhibited elevated infrared relative temperature at Yanggu (SI5) (*P* < 0.05) and reduced temperatures at Taiyang (EX-HN5), Quchi (LI11), and Waiqiu (GB36) (*P* < 0.01 for all) (**Table 4**).

**Table 4:** Comparison of infrared relative temperatures of acupoints between the MDD group and the HC group [Mean ± SD, m (Q1, Q3)].

Acupoints		HC group (n=40)	MDD group (n=60)	*t*/*z*	*P* value
Face	Yintang (GV29)	-0.09 ± 0.25	-0.12 ± 0.29	0.53	0.601
	Shuigou (GV26)	-0.20 (-0.76, 0.20)	-0.33 (-0.95, 0.32)	-0.29	0.770
	Taiyang (EX-HN5)	-0.01 ± 0.53	-0.42 ± 0.60	3.42	<0.001^**^
Medial upper limb	Chize (LU5)	0.68 ± 0.53	0.51 ± 0.45	1.76	0.081
	Yuji (LU10)	1.00 (-0.45, 1.69)	0.89 (-0.26, 1.41)	-0.58	0.559
	Neiguan (PC6)	0.55 (0.20, 0.88)	0.53 (0.27, 0.86)	-0.27	0.789
	Shenmen (HT7)	0.19 ± 0.87	0.13 ± 0.93	0.33	0.744
	Tongli (HT4)	0.36 ± 0.69	0.08 ± 0.80	1.83	0.071
	Shaohai (HT3)	-0.83 ± 0.68	-0.87 ± 0.59	0.33	0.745
Lateral upper limb	Quchi (LI11)	0.27 ± 0.52	-0.06 ± 0.63	2.76	0.007^**^
	Hegu (LI4)	0.87 (0.28, 1.23)	1.11 (0.53, 1.85)	-1.83	0.067
	Pianli (LI6)	0.06 ± 0.50	0.18 ± 0.51	-1.15	0.251
	Yanggu (SI5)	-0.55 ± 1.00	-0.06 ± 1.22	-2.10	0.038^*^
	Zhizheng (SI7)	-0.52 (-0.96, -0.27)	-0.39 (-0.68, 0.00)	-1.82	0.069
Medial lower limb	Sanyinjiao (SP6)	-0.28 ± 0.49	-0.28 ± 0.57	-0.02	0.985
	Yinlingquan (SP9)	0.05 ± 0.49	0.18 ± 0.51	-1.34	0.185
	Taixi (KI3)	-0.13 ± 0.76	-0.08 ± 1.08	-0.26	0.794
	Zhaohai (KI6)	0.59 ± 1.06	0.67 ± 1.41	-0.29	0.769
Lateral lower limb	Zusanli (ST36)	0.19 ± 0.44	0.35 ± 0.39	-1.95	0.054
	Fenglong (ST40)	0.28 ± 0.27	0.17 ± 0.30	1.97	0.052
	Jiexi (ST41)	0.68 ± 0.92	0.73 ± 1.21	-0.21	0.831
	Yanglingquan (GB34)	0.22 ± 0.46	0.29 ± 0.49	-0.79	0.434
	Waiqiu (GB36)	0.11 ± 0.38	-0.13 ± 0.30	3.44	<0.001^**^
	Qiuxu (GB40)	0.50 ± 1.17	0.34 ± 1.51	0.56	0.574
	Shenmai (BL62)	0.33 ± 1.25	0.15 ± 1.67	0.58	0.563
	Taichong (LR3)	0.17 ± 1.38	0.26 ± 1.79	-0.28	0.783
	Xingjian (LR2)	-0.23 ± 1.78	-0.32 ± 2.29	0.21	0.837

Data are presented as mean ± SD for normally distributed variables, or as M (Q1, Q3) for variables exhibiting non-normal distributions. Group comparisons were performed using the independent-samples t-test (*t*) for parametric data and the Mann-Whitney U test (*z*) for non-parametric data. ^*^P < 0.05, ^**^P < 0.01.

### 3.3 Correlation analysis

Further analysis was conducted to investigate whether there was a correlation between infrared relative temperature at Taiyang (EX-HN5), Quchi (LI11), Yanggu (SI5), and Waiqiu (GB36) and depression severity (n = 100). SDS scores were used as the reference index for evaluating depression severity.

**Figure 2:**
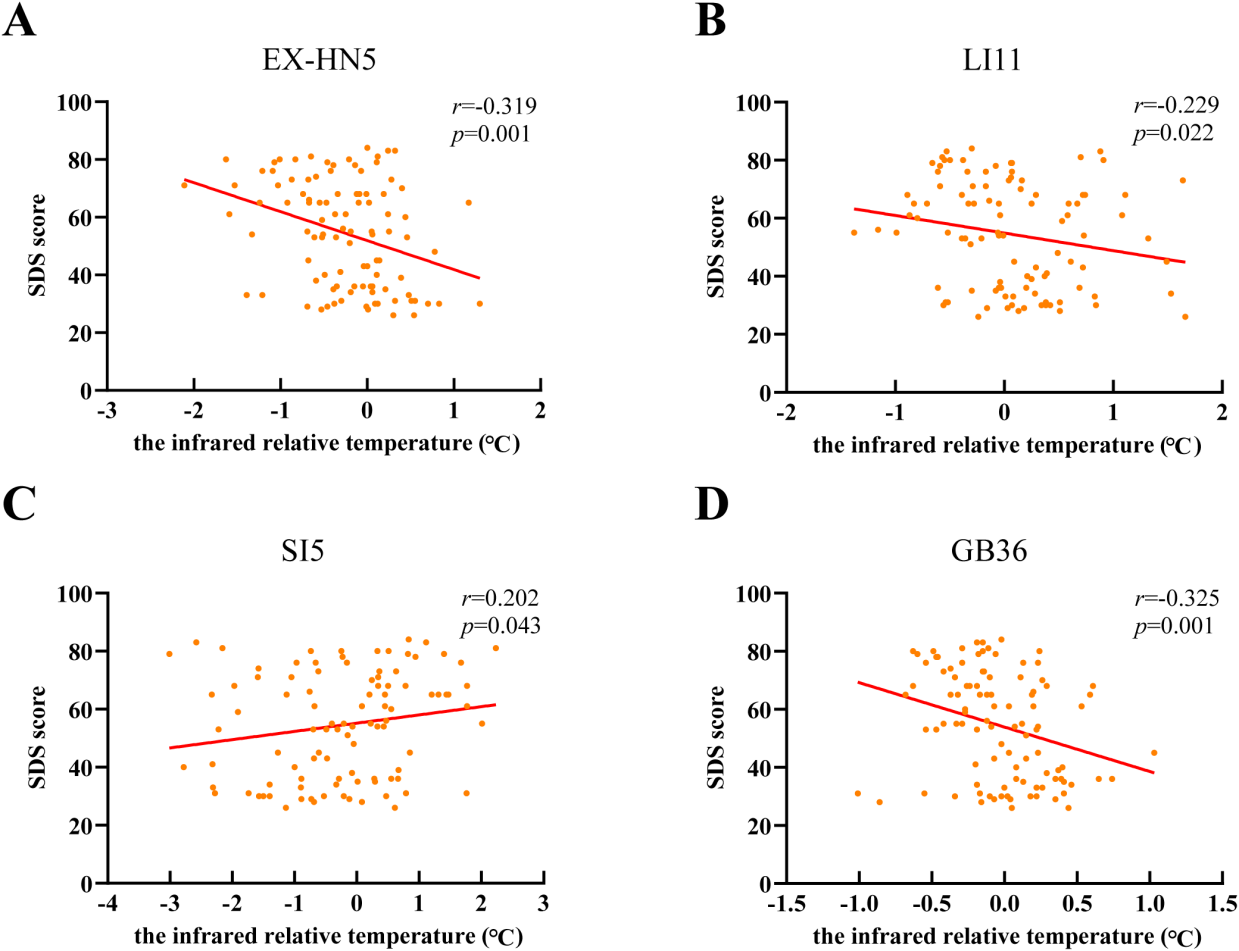
Correlation analysis between infrared relative temperature of four key acupoints and depression severity. The scatter plots display data from the combined cohort (n = 100, including adolescents with MDD and HCs). The solid red line represents the linear regression line of best fit. Correlation coefficients (*r*) and *P* values were calculated using Pearson’s correlation analysis. **(A)** Infrared relative temperature of Taiyang (EX-HN5) was negatively correlated with SDS score (*P* = 0.001, *r* = -0.319). **(B)** Infrared relative temperature at Quchi (LI11) was negatively correlated with SDS score (*P* = 0.022, *r* = -0.229). **(C)** Infrared relative temperature at Yanggu (SI5) was positively correlated with SDS score (*P* = 0.043, *r* = 0.202). **(D)** Infrared relative temperature at Waiqiu (GB36) was negatively correlated with SDS score (*P* = 0.001, *r* = -0.325).

The Pearsons rank coefficient r was calculated to indicate correlations. Pearson’s correlation coefficients were interpreted as follows: 0 < |r| ≤ 0.3 indicated a weak correlation; 0.3 < |r| ≤ 0.7 indicated a moderate correlation; and 0.7 < |r| ≤1 indicated a strong correlation (36). As shown in **Figure 2**, the infrared relative temperature at Taiyang (EX-HN5) and Waiqiu (GB36) showed a moderate anticorrelation with SDS scores (*P* = 0.001, *r* = -0.319; *P* = 0.001, *r* = -0.325). Although the infrared relative temperature at Quchi (LI11) also showed a negative correlation with SDS scores, the correlation was weak (*P* = 0.022, *r* = -0.229). Meanwhile, the infrared relative temperature at Yanggu (SI5) demonstrated a weak positive correlation with SDS scores (*P* = 0.022, *r* = -0.229; *P* = 0.043, *r* = 0.202).

### 3.4 Establishment of a diagnostic model for depression severity of adolescents

**Figure 3:**
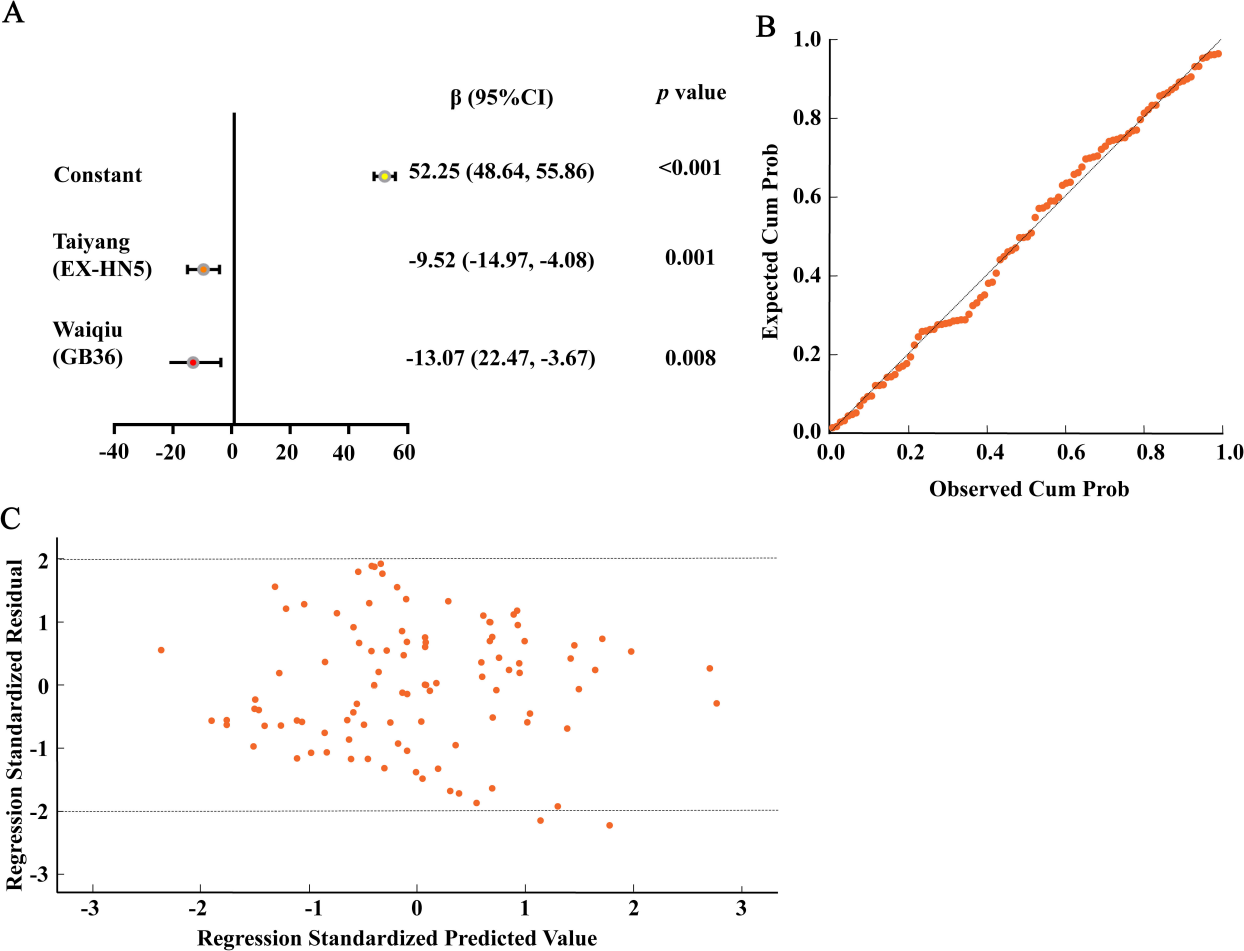
Statistical validation and parameter estimation of the multivariate linear regression model for predicting depression severity. **(A)** Forest plot of the final regression model predicting depression severity. Data are presented as regression coefficients (β) and 95% confidence intervals (CIs). **(B)** Normal P-P plot of regression-standardized residuals. **(C)** Scatterplot of regression-standardized residuals versus standardized predicted values..

Only variables that were significant in the correlation analysis (*P* ≤ 0.05) were selected for multiple linear regression analysis. Before constructing the model, a collinearity analysis was conducted on all independent variables. The tolerance of each variable was greater than 0.1, and the variance inflation factor (VIF) was less than 10, showing no obvious multicollinearity among the variables. Infrared relative temperatures at Taiyang (EX-HN5), Quchi (LI11), Yanggu (SI5), and Waiqiu (GB36) were used as independent variables, and the SDS score, as an evaluation index of depression severity, was deemed as the dependent variable in the multiple linear regression analysis. The results showed that the diagnostic parameters mainly includes Taiyang (EX-HN5) (β = -9.52, *P* = 0.001) and Waiqiu (GB36) (β = -13.07, *P* = 0.008). We combined the infrared relative temperature atTaiyang (EX-HN5) and Waiqiu (GB36) to build a diagnostic model for depression severity of adolescents: Y = 52.25-9.52*T_EX-HN5_-13.07*T_GB36_. The model explained 21.5% of the variance in depression severity among adolescents. F = 6.510, *P* < 0.01, indicating that the regression coefficients have statistical significance. Detailed results are shown in **Table 5** and **Figure 3A**. For distribution of residuals, see **Figures 3B, C**. The Probability-Probability (P-P) plot and scatterplot of the standard residuals showed that the assumptions of normality, linearity, and homoscedasticity were reasonably met.

**Table 5:** Multiple linear regression analysis identifying independent acupoint thermal predictors for depression severity in adolescents.

	β, 95% CI	*t*	*P* value	Collinearity diagnostics	
				Tolerance	VIF
Constant	52.25(48.64, 55.86)	28.36	<0.001^***^	/	/
Taiyang (EX-HN5)	-9.52(-14.97, -4.08)	-3.43	0.001^**^	1.026	0.975
Waiqiu (GB36)	-13.07(-22.47, -3.67)	-2.73	0.008^**^	1.047	0.955
R^2^	0.215				
F	6.510 (*P* < 0.01)				

The dependent variable is the clinical depression severity (SDS score). CI, confidence interval; VIF, variance inflation factor. The symbol “/” denotes not applicable. ^**^P < 0.01, ^***^P < 0.001.

### 3.5 Establishment of the diagnostic model for adolescent MDD and its validation

A binary logistic regression analysis was performed with MDD status (“no” = 0, “yes” = 1) as the dependent variable. The four predictive variables (infrared relative temperatures at Taiyang [EX-HN5), Quchi (LI11), Yanggu (SI5), and Waiqiu (GB36)] identified through correlation analysis were included as independent variables in a multivariable binary logistic regression. The results revealed that infrared relative temperatures at Taiyang (EX-HN5) and Waiqiu (GB36) were independent risk factors for MDD in adolescents (β = -1.44, *P* = 0.002; β = -2.19, *P* = 0.003), while the infrared relative temperature at Yanggu (SI5) emerged as a protective factor for MDD in adolescents (β = 0.45, *P* = 0.037). The infrared relative temperature at Quchi (LI11) displayed no significant statistical meaning (*P* > 0.05) (**Table 6**).

**Table 6:** Binary logistic regression model for predicting MDD diagnosis in adolescents based on acupoint temperatures.

Variable	β	S.E	Z	*P* value	OR (95%CI)	Collinearity diagnostics	
						Tolerance	VIF
Taiyang(EX-HN5)	-1.44	0.46	-3.15	0.002^**^	0.24 (0.10-0.58)	0.975	1.026
Yanggu(SI5)	0.45	0.21	2.08	0.037^*^	1.56 (1.03-2.38)	0.711	1.407
Waiqiu(GB36)	-2.19	0.75	-2.94	0.003^**^	0.11 (0.03-0.48)	0.955	1.047

The dependent variable is the diagnostic status (MDD vs. HC). S.E, standard error; OR, odds ratio; CI, confidence interval; VIF, variance inflation factor. ^*^P < 0.05, ^**^P < 0.01.

According to the partial regression coefficient β of each independent risk factor and constant term in the logistic regression results, the logistic regression equation was obtained, and the diagnostic model for adolescent MDD was constructed: P = e^x^/(1+e^x^), x = 0.22-1.14*T_EX-HN5_+0.45*T_SI5_-2.19*T_GB36_, where P represents the predicted probability of MDD in adolescents.

**Figure 4:**
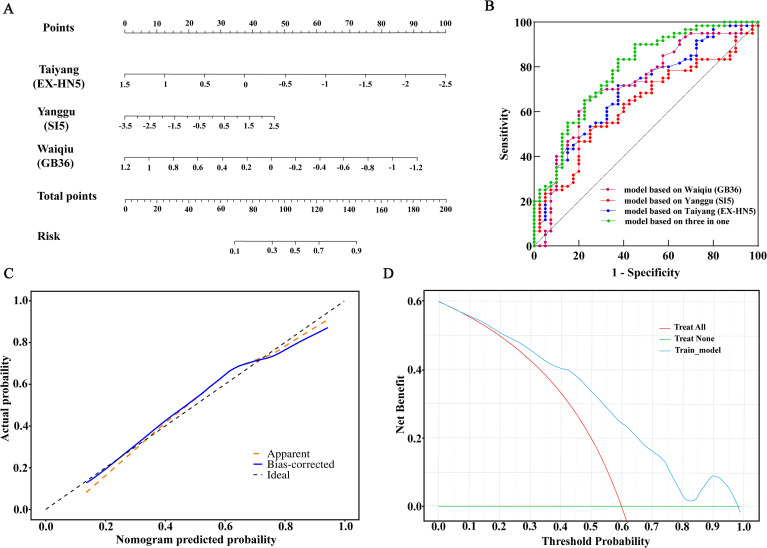
Development and performance validation of the diagnostic nomogram for MDD in adolescents. **(A)** Diagnostic nomogram for predicting the probability of MDD, incorporating the infrared relative temperatures of three key acupoints (Taiyang (EX-HN5), Yanggu (SI5), and Waiqiu (GB36)). To utilize the nomogram, locate the specific value for each acupoint on its respective axis, draw a vertical line upward to the “Points” axis to assign a score, add the scores from all three variables, and locate the final sum on the “Total points” axis. A vertical line drawn downward to the “Risk” axis indicates the predicted probability of MDD. **(B)** Receiver ROC curves comparing the combined nomogram model against single-acupoint models. **(C)** Calibration curves assessing the agreement between predicted and observed probabilities. The bias-corrected performance was evaluated using bootstrap resampling. **(D)** DCA evaluating the clinical utility of the nomogram compared to the treat-all and treat-none strategies..

In order to make the model more intuitive and more convenient for clinical application, the above logistic regression equation was drawn into a nomogram for visualization (**Figure 4A**). The nomogram is used as follows: for each variable (infrared relative temperatures at Taiyang (EX-HN5), Yanggu (SI5), and Waiqiu (GB36)), draw an upward vertical line to the “Points” bar to calculate points. Then, add the points for each variable and locate this sum on the “Total Points” axis. Draw a downward vertical line from the “Total Points” line to calculate the estimated probability of MDD in adolescents. Higher total scores on the x-axis correspond to an increased likelihood of MDD occurrence.

Waiqiu (GB36) showed the best discriminative ability (AUC = 0.7198; 95%CI: 0.6149 - 0.8247) among all 3 indicators (all *P* < 0.05), including Waiqiu (GB36) (AUC = 0.7198), Taiyang (EX-HN5) (AUC = 0.6952), and Yanggu (SI5) (AUC = 0.6313). The combined model incorporating Taiyang (EX-HN5), Yanggu (SI5), and Waiqiu (GB36) demonstrated superior discriminative performance (AUC = 0.785, 95% CI: 0.693 - 0.876, *P* < 0.001) compared to individual parameters (**Figure 4B**). The diagnostic model for adolescent MDD was internally validated using bootstrap resampling with 1000 iterations. The results showed a C-index of 0.752 (95% CI: 0.617 - 0.877), indicating good discriminative ability.

The results of the Hosmer-Lemeshow test were respectively, modeling set: *P* = 0.855, and there was no statistically significant difference between the risk prediction value and the actual observation value, indicating that the model has good prediction accuracy. The calibration curve of the model showed that the fitted curve (representing predicted probabilities) and the reference line (representing ideal calibration) had a high degree of coincidence, indicating that the model has good calibration for predicting fracture occurrence. Specifically, the predicted probabilities and the observed frequencies were respectively well aligned with the fitted curve and the reference line. The calibration curves are shown in **Figure 4C**.

The decision curves indicated that patients could obtain more clinical net benefits across a wide range of threshold probabilities compared to both treat-all and treat-none schemes. The clinical impact curves also indicated that the model had meaningful clinical usefulness (**Figure 4D**).

## 4 Discussion

The application of IRT to investigate acupoint temperature marks a significant advancement in the biophysical research of acupoints (37). During temperature measurement, it is typical for the same IRT image to be analyzed by various investigators or by the same investigator at different times (34, 38). Prior to this study, a standardized instrument was needed for characteristics and reliability assessment to ensure reproducible results. However, to date, no study has reported a reliability analysis of IRT in adolescents with MDD. In this study, 27 acupoints located on the face and limbs were selected based on the results of data mining. To assess reliability, the Bland-Altman plot and ICC were utilized. Our results demonstrated excellent reliability, with inter- and intra-investigator ICC values ranging from 0.809 to 0.999. Bland-Altman analysis corroborated the ICC findings, with approximately 92.6%–100% of data points falling within the 95% limits of agreement, thereby indicating that the consistency of our study data was validated. The results of the consistency analysis can be used as quality control for subsequent larger diagnostic studies. In addition, strict control of the testing environment, subjects, and acupoint temperature analysis further ensured the objectivity of the final test results.

In recent years, the role of human temperature in MDD has garnered increasing scientific attention. Alterations in core body temperature may induce activation of thermosensitive transient receptor potential (TRP) channels, whereby cation influx converts thermal signals into electrical impulses, subsequently enhancing neuronal firing and potentially contributing to stress-associated depression (39). Additionally, blunted peripheral temperature reactivity has been identified as a characteristic feature in individuals with depression (40). However, a paucity of systematic investigations exists regarding skin acupoint temperature in neuroanatomical proximity. This study conducted a systematic temperature evaluation of 27 acupoints for MDD treatment. The findings confirmed that adolescents with MDD exhibit altered acupoint temperatures, specifically at Taiyang (EX-HN5), Quchi (LI11), Yanggu (SI5), and Waiqiu (GB36). Furthermore, the infrared relative temperatures at Taiyang (EX-HN5), Quchi (LI11), and Waiqiu (GB36) demonstrated negative correlations with depression severity, whereas that at Yanggu (SI5) exhibited positive correlations with depression severity. Taiyang (EX-HN5) is located at the boundary between the temporal, sphenoid, frontal, and parietal bones, where many nerves and blood vessels are clustered. Stimulation of Taiyang (EX-HN5) may indirectly stimulate the vegetative nerve endings near the acupoints, thus regulating the excitatory or inhibitory signals sent from the cerebral cortex (41). The deeper part of Taiyang (EX-HN5) corresponds to the temporal lobe, which is closely related to functions such as cognition, emotion, and memory (42, 43). Quchi (LI11), Yanggu (SI5), and Waiqiu (GB36) are located on the limbs. From the perspective of neuroanatomical proximity, changes in the temperature at these acupoints may modulate adjacent peripheral nerve terminals (e.g., the superficial peroneal nerve) (44). The dorsal root fibers of these nerves transmit afferent signals to the spinal cord, subsequently influencing central nervous system activity, which could mediate emotional-cognitive regulation (45, 46). Furthermore, sympathovagal imbalance may represent another underlying mechanism through which acupoint temperature reflects depressive pathophysiology (47).

This study proposes a novel application of acupoint infrared relative temperature as a predictive biomarker for MDD, establishing two non-invasive diagnostic models: a diagnostic model for adolescent depression severity and a diagnostic model for MDD in adolescents. Our study also examined the temperature of bilateral homonymous acupoints in adolescents with MDD, and no significant difference in temperature between the left and right sides was observed (**Supplementary Material 2**). Consequently, clinical implementation requires only unilateral acupoint infrared relative temperature data input into the diagnostic models to achieve either severity assessment or auxiliary diagnosis of adolescent MDD. This approach offers two key advantages: first, it significantly reduces detection time, thereby enhancing feasibility for large-scale mental health screenings; and second, it prevents data loss caused by positional constraints during testing or unilateral skin lesions, thereby ensuring data integrity.

In contrast to existing diagnostic models for MDD (e.g., multi-task attentional temporal convolutional networks designed for MDD recognition) (48), our IRT-based approach demonstrates distinct advantages in clinical practicality. Specifically, the measurement of acupoint temperatures using IRT does not require complex computational infrastructure or large-scale datasets, enabling simpler operational procedures while maintaining comparable diagnostic accuracy (AUC = 0.785 vs. 0.720 reported in prior models). Importantly, our method uniquely integrates dual functionality: it not only assists in diagnosing MDD but also provides quantitative insights into depression severity through infrared relative temperature variations at key acupoints (e.g., Taiyang (EX-HN5) and Waiqiu (GB36)).

To fully appreciate the clinical implications of these findings, it is essential to situate our IRT-based approach and results within the broader landscape of objective psychiatric biomarkers and digital phenotyping. Traditionally, the diagnosis of MDD, particularly in adolescents, relies heavily on subjective self-reports and clinical interviews, which are inherently susceptible to recall bias, subjective interpretation, and communication barriers. Consequently, the field of precision psychiatry is experiencing a paradigm shift toward the establishment of objective and biologically anchored biomarkers. This shift encompasses a diverse array of measures, including neuroimaging, biochemical assays, and autonomic physiological metrics (49–51). In this context, we employed IRT-measured acupoint temperature as a non-invasive, quantifiable physiological biomarker that directly captures thermoregulatory dysfunction inherent to depressive pathology. The rapid evolution of psychiatric biomarkers has been significantly propelled by the advent of digital phenotyping. Notably, recent advances in wearable technology, such as wristband sensors, enable continuous and passive tracking of physiological parameters, including peripheral skin temperature, blood volume pulse, and electrodermal activity. These data streams have proven highly effective for classifying depression subtypes (52, 53). However, while wearable devices excel in capturing ambulatory time-series data, our IRT methodology introduces high-resolution, site-specific thermal measurements at anatomically defined acupoints, offering a different level of granularity. By linking acupoint temperature to traditional meridian theory and neuroanatomical correlates, our work bridges ancient medical knowledge with modern biophysical measurement, offering a unique conceptual framework that complements data-driven digital phenotyping approaches. Future developments in this field should focus on integrating IRT-based acupoint evaluations with continuous physiological data from wearable devices. This synthesis promises to establish a robust and comprehensive digital phenotype for mood disorders. By merging the static, localized thermal profiles obtained through IRT with the dynamic tracking of temperature, autonomic arousal, and sleep cycles provided by wearables, researchers can simultaneously capture spatial and temporal physiological changes. The integration of these complementary datasets holds significant potential to enhance diagnostic accuracy and facilitate personalized therapeutic monitoring. Ultimately, such a multimodal spatiotemporal strategy advances the development of precision psychiatry.

The innovation of these models lies in their application of IRT to evaluate the temperature of MDD-related acupoints, offering distinct advantages, including non-invasiveness, absence of radiation exposure, ease of data collection, and low-cost. Compared to traditional psychiatric evaluations, these objective metrics circumvent biases associated with adolescent self-reporting, thereby significantly enhancing participant compliance. Furthermore, streamlined parameter integration reduces clinical data collection burdens, improving applicability in primary care settings. Clinically, the diagnostic model for MDD in adolescents is visualized as a nomogram, enabling clinicians to rapidly estimate MDD probability by inputting acupoint infrared relative temperature values. This tool enhances early screening and preventive strategies for adolescent MDD, particularly in school-based mental health screenings and preliminary diagnostics in primary healthcare institutions and public health agencies, thereby partially addressing the shortage of specialized psychiatric resources. Concurrently, longitudinal monitoring of acupoint temperature may be used to monitor treatment efficacy; for instance, changes in acupoint temperature may be associated with symptom improvement following acupuncture or pharmacotherapy. Prospectively, we aim to develop these non-invasive diagnostic models into digital apps integrated with cloud-based platforms to deliver real-time intervention recommendations.

Several limitations of this study should be acknowledged. First, the relatively small sample size may constrain the model’s predictive potential. Internal validation via bootstrapping yielded a C-index of 0.752, slightly lower than the original AUC of 0.785. Although this decline (Δ = 0.033) is minor, it suggests a degree of model optimism and potential overfitting, indicating that discriminative performance may diminish in unseen populations. Therefore, external validation across independent, multi-center cohorts is necessary to confirm generalizability. Second, the current study lacks the inclusion of other established diagnostic factors. To further optimize predictive accuracy, subsequent studies should incorporate multi-modal data, including biochemical or neuroimaging markers, thereby enabling the development of more robust diagnostic models for adolescent MDD. Third, the lack of detailed records regarding antidepressant use during IRT assessments introduces potential confounding. Psychotropic drugs, particularly selective serotonin reuptake inhibitors, can influence autonomic function and peripheral thermoregulation, potentially confounding acupoint temperature readings. Future investigations must recruit medication-naïve participants or strictly stratify by pharmacological history to more accurately isolate disease-related effects. Finally, as a preliminary investigation, this study prioritized diagnostic homogeneity by excluding adolescents with comorbid psychiatric conditions, limiting comparisons to MDD versus HCs. Thus, it remains unclear whether the observed temperature variations are specific to MDD or represent transdiagnostic physiological features. To firmly establish discriminant validity and facilitate differential diagnosis, subsequent studies should include clinical control groups with distinct psychiatric conditions, such as bipolar disorder or anxiety disorders.

## 5 Conclusion

In conclusion, our study showed that acupoint temperatures at Taiyang (EX-HN5), Quchi (LI11), Yanggu (SI5), and Waiqiu (GB36) were altered in adolescents with MDD. The infrared relative temperatures at Taiyang (EX-HN5) and Waiqiu (GB36) exhibited moderate negative correlations with depression severity. Based on these findings, we developed two predictive models: a diagnostic model for depression severity in adolescents and a diagnostic model for adolescent MDD. These models provide novel methodological perspectives and reliable tools for early prevention and clinical diagnosis of adolescent MDD, highlighting their significant potential for clinical application in MDD management.

## Data Availability

The original contributions presented in the study are included in the article/**Supplementary Material**. Further inquiries can be directed to the corresponding authors.
