# Supplementary material for: Acupoint temperature as a biomarker: infrared thermography in the diagnosis of adolescents with major depressive disorder
Source: Front Psychiatry. 2026 Apr 29;17:1806676. doi: 10.3389/fpsyt.2026.1806676 (PMC13168048; doi:10.3389/fpsyt.2026.1806676)
Supplement: Supplementary file 1 [file SupplementaryFile1.docx]

# *Reliability assessment of infrared thermography for measuring acupoint temperatures in adolescents with MDD*

# Materials and methods

## Participants

## From May 2022 to September 2023, adolescents with Major Depressive Disorder (MDD) were recruited from the Third Affiliated Hospital of Zhejiang Chinese Medical University, Hangzhou First People’s Hospital, and Tongde Hospital of Zhejiang Province, and diagnosed based on International Classification of Diseases-10 (ICD-10) by psychiatrists with 20 years of working experience. The Self-rating Depression Scale (SDS) was completed by all subjects to assess the severity of depressive disorder. Only patients with SDS score greater than 53 were included in the study. The exclusion criteria included: (1) Severe anxiety, schizophrenia, or other serious mental illnesses; (2) There are pigmentation, redness, infection or scarring on the skin at the site of detection. (3) Participants with severe systemic diseases and their complications, serious infections, and other major medical conditions; (4) Participants who were pregnant, lactating, during, or in proximity (±2 days) to their menstruation or ovulation; (5) Participants with a body temperature ≥37.3°C; (6) Participants who did not complete the infrared thermography (IRT) measurement. All subjects volunteered to participate in this study provided assent, while parents or guardians provided informed consent. Investigator agreement was assessed in a subset of patients (n = 27) using PASS 15.0.5 software (power = 85%, α = 0.05, ρ0 = 0.60, ρ1 = 0.85), accounting for a 20% attrition rate.

## Acquisition of IRT images

IRT image acquisition was performed following the standardized protocol detailed in Materials and methods.

## The detection acupoints and analytical method

The detection acupoints and analytical methodology strictly adhered to the experimental procedures delineated in Materials and methods.

## Statistical Analysis

SPSS 25.0 (IBM SPSS Statistics for Windows, USA), GraphPad Prism 8.0.2 (GraphPad Prism, USA.) were used for statistical analysis and graphing. All normally distributed data are presented as Mean ± SD, and non-normally distributed data are presented as M (Q₁, Q₃). Normally distributed data (assessed via Shapiro-Wilk test) were analyzed using paired t-tests, while non-parametric Wilcoxon rank-sum tests were applied to skewed distributions. Significance was set at P < 0.05. In terms of reliability, ICC measures both correlation and agreement between measurements (1). ICC values allowed the classification of the agreement into four classes: poor (ICC < 0.40), fair (0.4 ≤ ICC < 0.60), good (0.60 ≤ ICC < 0.75), and excellent (0.75 ≤ ICC ≤ 1.00) (2). Furthermore, differences between two separate investigator and intra-investigator were graphically displayed using Bland-Altman plots. Drawing from prior research (3), the interval of confidence was set at 95%.

# Result

To test inter- and intra-investigator reliability, infrared relative temperature of acupoint was performed by two investigators at two different time points. There were no significant differences between the two investigators (Table 1), Meanwhile, no statistically significant differences were found between the investigators at any point (Table 2, 3).

Both intra- and inter-investigator reliability were excellent, with intraclass correlation coefficient (ICC) values ranging from 0.809 to 0.999 (Table 4). Bland-Altman analysis further confirmed consistency, with 92.6–100% of data points within the 95% limits of agreement (Fig. 1-15).

**References**

1. Koo TK, Li MY. A guideline of selecting and reporting intraclass correlation coefficients for reliability research. *J Chiropr Med* (2016) 15:155-63. doi: 10.1016/j.jcm.2016.02.012

2. Pérez Boal E, Martin-Villa C, Becerro de Bengoa Vallejo R, Losa Iglesias ME, Trevissón Redondo B, Casado Hernández I, et al. Intra and inter-observer reliability and repeatability of metatarsus adductus angle in recreational football players: A concordance study. *J Clin Med* (2021) 11:2043. doi: 10.3390/jcm11072043

3. Lou JL, JiangYL, Hu HT, Li XY, Zhang YJ, Fang JQ. Intrarater and interrater reliability of infrared image analysis of forearm acupoints before and after moxibustion. *Evid Based Complement Alternat Med* (2020) 6328756. doi: 10.1155/2020/6328756

Table 1 The infrared relative temperature of acupoint of 2 investigators in one sessions [Mean ± SD, M (Q1, Q3)]

| Acupoints | | Investigator 1 (n=27) | Investigator 2 (n=27) | *t*/*z* | *P* value |
| --- | --- | --- | --- | --- | --- |
| Face | Yintang (GV29) | -0.11 (-0.30, 0.16) | -0.10 (-0.29, 0.11) | -0.970 | 0.332 |
|  | Shuigou (GV26) | -0.43 ± 1.15 | -0.42 ± 1.13 | -0.88 | 0.385 |
|  | Taiyang (EX-HN5) | -0.44 ± 0.78 | -0.43 ± 0.75 | -0.50 | 0.625 |
| Medial upper limb | Chize (LU5) | 0.49 ± 0.59 | -0.48 ± 0.58 | 1.30 | 0.207 |
|  | Yuji(LU10) | 0.23 (-1.24, 1.66) | 0.22 (-1.14, 1.06) | -1.33 | 0.182 |
|  | Neiguan (PC6) | 0.35 ± 0.60 | 0.33 ± 0.60 | 1.93 | 0.065 |
|  | Shenmen (HT7) | -0.14 ± 1.24 | -0.15 ± 1.24 | 0.84 | 0.408 |
|  | Tongli (HT4) | 0.06 ± 0.88 | 0.04 ± 0.89 | 1.38 | 0.18 |
|  | Shaohai (HT3) | -0.83 (-1.07, -0.40) | -0.83 (-1.03, -0.37) | -0.87 | 0.385 |
| Lateral upper limb | Quchi (LI11) | -0.10 ± 0.83 | -0.09 ± 0.81 | -0.58 | 0.565 |
|  | Hegu (LI4) | 0.67 ± 1.12 | 0.65 ± 1.12 | 1.95 | 0.062 |
|  | Pianli (LI6) | 0.13 ± 0.53 | 0.13 ± 0.58 | 0.05 | 0.963 |
|  | Yanggu (SI5) | -0.31 ± 1.34 | -0.30 ± 1.29 | -0.97 | 0.344 |
|  | Zhizheng (SI7) | -0.39 (-0.76, -0.01) | -0.46 (-0.89, 0.00) | -0.03 | 0.980 |
| Medial lower limb | Sanyinjiao (SP6) | -0.07 ± 0.75 | -0.08 ± 0.75 | 0.17 | 0.863 |
|  | Yinlingquan (SP9) | 0.19 ± 0.52 | 0.18 ± 0.50 | 0.61 | 0.546 |
|  | Taixi (KI3) | -0.74 ± 0.23 | -0.82 ± 0.22 | 0.41 | 0.687 |
|  | Zhaohai (KI6) | 0.59 ± 1.54 | 0.57 ± 1.54 | 2.03 | 0.053 |
| Lateral lower limb | Zusanli (ST36) | 0.39 ± 0.43 | 0.39 ± 0.43 | -0.58 | 0.566 |
|  | Fenglong(ST40) | 0.13 ± 0.33 | 0.14 ± 0.33 | -0.49 | 0.63 |
|  | Jiexi (ST41) | 0.73 ± 1.36 | 0.74 ± 1.34 | -0.75 | 0.463 |
|  | Yanglingquan (GB34) | 0.34 ± 0.52 | 0.34 ± 0.49 | 0.47 | 0.641 |
|  | Waiqiu (GB36) | -0.20 ± 0.27 | -0.20 ± 0.27 | 0.07 | 0.948 |
|  | Qiuxu (GB40) | 0.36 ± 1.66 | 0.35 ± 1.61 | 0.91 | 0.369 |
|  | Shenmai (BL62) | 0.23 (-0.93, 1.69) | 0.25 (-0.99, 1.69) | -0.65 | 0.516 |
|  | Taichong (LR3) | 0.20 ± 2.14 | 0.18 ± 2.12 | 1.55 | 0.134 |
|  | Xingjian (LR2) | -0.17 ± 2.36 | -0.19 ± 2.39 | 1.89 | 0.070 |

Table 2 Infrared relative temperature of acupoint of the investigator 1 in two sessions [Mean±SD, M (Q1, Q3)]

| Acupoints | | Investigator 1  Session 1(n=27) | Investigator 1  Session 2 (n=27) | *t*/*z* | *P* value |
| --- | --- | --- | --- | --- | --- |
| Face | Yintang (GV29) | -0.11 ± 0.39 | -0.11 ± 0.36 | 0.14 | 0.892 |
|  | Shuigou (GV26) | -0.43 ± 1.15 | -0.41 ± 1.10 | -0.55 | 0.591 |
|  | Taiyang (EX-HN5) | -0.44 ± 0.78 | -0.41 ± 0.75 | -1.14 | 0.264 |
| Medial upper limb | Chize (LU5) | 0.49 (0.14, 0.75) | 0.48 (0.16, 0.88) | -0.46 | 0.648 |
|  | Yuji (LU10) | 0.27 (-0.52, 1.59) | 0.27 (-0.52, 1.59) | -0.23 | 0.816 |
|  | Neiguan (PC6) | 0.35 ± 0.60 | 0.32 ± 0.55 | 1.14 | 0.264 |
|  | Shenmen (HT7) | -0.14 (-0.78, 0.61) | -0.18 (-1.03, 0.61) | -0.43 | 0.665 |
|  | Tongli (HT4) | 0.06 (-0.24, 0.80) | -0.03 (-0.58, 0.84) | -0.89 | 0.373 |
|  | Shaohai (HT3) | -0.83 (-1.07, -0.40) | -0.83 (-1.21, -0.41) | -0.45 | 0.656 |
| Lateral upper limb | Quchi (LI11) | -0.10 ± 0.83 | -0.10 ± 0.83 | -0.01 | 0.989 |
|  | Hegu (LI4) | 0.67 (0.12, 1.24) | 0.66 (0.16, 1.41) | -0.35 | 0.727 |
|  | Pianli (LI6) | 0.13 ± 0.53 | 0.10 ± 0.59 | 0.97 | 0.340 |
|  | Yanggu (SI5) | -0.31 (-1.13, 0.51) | -0.19 (-1.18, 0.74) | -0.55 | 0.580 |
|  | Zhizheng (SI7) | -0.39 ± 0.63 | -0.41 ± 0.65 | 0.49 | 0.626 |
| Medial lower limb | Sanyinjiao (SP6) | -0.07 ± 0.75 | -0.08 ± 0.79 | 0.35 | 0.733 |
|  | Yinlingquan (SP9) | 0.19 ± 0.52 | 0.19 ± 0.54 | 0.07 | 0.948 |
|  | Taixi (KI3) | -0.07 ± 1.20 | -0.08 ± 1.17 | 0.24 | 0.811 |
|  | Zhaohai(KI6) | 0.59 ± 1.54 | 0.56 ± 1.50 | 1.53 | 0.138 |
| Lateral lower limb | Zusanli (ST36) | 0.39 ± 0.43 | 0.38 ± 0.46 | 0.28 | 0.779 |
|  | Fenglong (ST40) | 0.13 ± 0.33 | 0.11 ± 0.31 | 0.99 | 0.332 |
|  | Jiexi (ST41) | 0.73 (0.05, 1.56) | 0.72 (0.09, 1.39) | 0.00 | 1.000 |
|  | Yanglingquan (GB34) | 0.34 (0.07, 0.76) | 0.36 (0.03, 0.66) | -0.89 | 0.374 |
|  | Waiqiu (GB36) | -0.20 ± 0.27 | -0.20 ± 0.26 | -0.15 | 0.880 |
|  | Qiuxu (GB40) | 0.36 ± 1.66 | 0.38 ± 1.55 | -0.60 | 0.552 |
|  | Shenmai (BL62) | 0.23 ± 1.80 | 0.22 ± 1.70 | 0.30 | 0.763 |
|  | Taichong (LR3) | 0.20 (-1.25, 1.34) | 0.15 (-1.21, 1.29) | -0.81 | 0.416 |
|  | Xingjian (LR2) | -0.17 ± 2.36 | -0.14 ± 2.31 | -1.44 | 0.161 |

Table 3 Infrared relative temperature of acupoint of the investigator 2 in two sessions [Mean ± SD, M (Q1, Q3)]

| Acupoints | | Investigator 2  Session 1 (n=27) | Investigator 2  Session 2 (n=27) | *t*/*z* | *P* value |
| --- | --- | --- | --- | --- | --- |
| Face | Yintang (GV29) | -0.10 ± 0.38 | -0.06 ± 0.34 | -1.60 | 0.121 |
|  | Shuigou (GV26) | -0.42 ± 1.13 | -0.39 ± 1.04 | -0.92 | 0.364 |
|  | Taiyang (EX-HN5) | -0.43 ± 0.75 | -0.39 ± 0.70 | -1.37 | 0.183 |
| Medial upper limb | Chize(LU5) | 0.48 (0.13, 0.77) | 0.45 (0.13, 0.72) | -0.97 | 0.330 |
|  | Yuji (LU10) | 0.22 (-1.14, 1.62) | 0.24 (-1.01, 1.58) | -0.82 | 0.413 |
|  | Neiguan (PC6) | 0.33 ± 0.60 | 0.33 ± 0.60 | -0.07 | 0.943 |
|  | Shenmen (HT7) | -0.15 ± 1.24 | -0.13 ± 1.19 | -0.87 | 0.394 |
|  | Tongli (HT4) | 0.04 (-0.28, 0.76) | 0.03 (-0.22, 0.59) | -0.55 | 0.580 |
|  | Shaohai (HT3) | -0.83 ± 0.66 | -0.84 ± 0.64 | 0.56 | 0.583 |
| Lateral upper limb | Quchi (LI11) | -0.09 (-0.44, 0.12) | -0.08 (-0.44, 0.17) | -0.60 | 0.548 |
|  | Hegu (LI4) | 0.65 ± 1.12 | 0.68 ± 1.06 | -1.02 | 0.316 |
|  | Pianli (LI6) | 0.13 (-0.21, 0.41) | 0.12 (-0.28, 0.51) | -0.42 | 0.674 |
|  | Yanggu (SI5) | -0.30 ± 1.29 | -0.31 ± 1.20 | 0.69 | 0.497 |
|  | Zhizheng (SI7) | -0.46 ± 0.57 | -0.44 ± 0.61 | -0.90 | 0.376 |
| Medial lower limb | Sanyinjiao (SP6) | -0.08 ± 0.75 | -0.09 ± 0.69 | 0.44 | 0.664 |
|  | Yinlingquan (SP9) | 0.18 ± 0.50 | 0.18 ± 0.51 | 0.00 | 1.000 |
|  | Taixi (KI3) | -0.08 (-0.97, 0.88) | -0.11 (-0.86, 0.77) | -1.09 | 0.274 |
|  | Zhaohai (KI6) | 0.57 ± 1.54 | 0.55 ± 1.46 | 0.50 | 0.620 |
| Lateral lower limb | Zusanli (ST36) | 0.39 ± 0.43 | 0.37 ± 0.40 | 0.90 | 0.378 |
|  | Fenglong (ST40) | 0.14 ± 0.33 | 0.13 ± 0.32 | 0.23 | 0.823 |
|  | Jiexi (ST41) | 0.74 ± 1.34 | 0.75 ± 1.27 | -0.52 | 0.609 |
|  | Yanglingquan (GB34) | 0.34 (0.03, 0.67) | 0.34 (0.11, 0.58) | -0.39 | 0.693 |
|  | Waiqiu (GB36) | -0.20 ± 0.27 | -0.22 ± 0.24 | 0.70 | 0.489 |
|  | Qiuxu (GB40) | 0.35 ± 1.61 | 0.38 ± 1.52 | -1.16 | 0.259 |
|  | Shenmai (BL62) | 0.25 (-0.99, 1.69) | 0.24 (-1.06, 1.55) | -0.35 | 0.727 |
|  | Taichong (LR3) | 0.18 (-1.31, 1.29) | 0.18 (-1.09, 1.37) | -0.08 | 0.933 |
|  | Xingjian (LR2) | -0.19 (-2.33, 1.61) | -0.15 (-2.17, 1.58) | -1.12 | 0.264 |

Table 4 Inter- and intra-investigator reliability of infrared relative temperature of acupoint

| Acupoints | | Interrater reliability  Session 1 | | | Intrarater reliability  Session 1 | | Intrarater reliability  Session 2 | |
| --- | --- | --- | --- | --- | --- | --- | --- | --- |
|  |  | ICC | Scale | | ICC | Scale | ICC | Scale |
| Face | Yintang (GV29) | 0.990 | | Excellent | 0.956 | Excellent | 0.941 | Excellent |
|  | Shuigou (GV26) | 0.999 | | Excellent | 0.994 | Excellent | 0.992 | Excellent |
|  | Taiyang (EX-HN5) | 0.989 | | Excellent | 0.987 | Excellent | 0.984 | Excellent |
| Medial upper limb | Chize (LU5) | 0.991 | | Excellent | 0.973 | Excellent | 0.971 | Excellent |
|  | Yuji (LU10) | 0.998 | | Excellent | 0.978 | Excellent | 0.997 | Excellent |
|  | Neiguan (PC6) | 0.992 | | Excellent | 0.970 | Excellent | 0.976 | Excellent |
|  | Shenmen (HT7) | 0.998 | | Excellent | 0.980 | Excellent | 0.995 | Excellent |
|  | Tongli (HT4) | 0.997 | | Excellent | 0.941 | Excellent | 0.958 | Excellent |
|  | Shaohai (HT3) | 0.997 | | Excellent | 0.972 | Excellent | 0.979 | Excellent |
| Lateral upper limb | Quchi (LI11) | 0.997 | | Excellent | 0.987 | Excellent | 0.993 | Excellent |
|  | Hegu (LI4) | 0.998 | | Excellent | 0.992 | Excellent | 0.991 | Excellent |
|  | Pianli (LI6) | 0.989 | | Excellent | 0.970 | Excellent | 0.980 | Excellent |
|  | Yanggu (SI5) | 0.998 | | Excellent | 0.889 | Excellent | 0.995 | Excellent |
|  | Zhizheng (SI7) | 0.809 | | Excellent | 0.983 | Excellent | 0.980 | Excellent |
| Medial lower limb | Sanyinjiao (SP6) | 0.996 | | Excellent | 0.987 | Excellent | 0.983 | Excellent |
|  | Yinlingquan (SP9) | 0.993 | | Excellent | 0.976 | Excellent | 0.977 | Excellent |
|  | Taixi (KI3) | 0.997 | | Excellent | 0.994 | Excellent | 0.990 | Excellent |
|  | Zhaohai (KI6) | 0.999 | | Excellent | 0.997 | Excellent | 0.994 | Excellent |
| Lateral lower limb | Zusanli (ST36) | 0.993 | | Excellent | 0.971 | Excellent | 0.962 | Excellent |
|  | Fenglong (ST40) | 0.982 | | Excellent | 0.946 | Excellent | 0.945 | Excellent |
|  | Jiexi (ST41) | 0.999 | | Excellent | 0.992 | Excellent | 0.993 | Excellent |
|  | Yanglingquan (GB34) | 0.992 | | Excellent | 0.977 | Excellent | 0.967 | Excellent |
|  | Waiqiu (GB36) | 0.977 | | Excellent | 0.931 | Excellent | 0.894 | Excellent |
|  | Qiuxu (GB40) | 0.999 | | Excellent | 0.994 | Excellent | 0.994 | Excellent |
|  | Shenmai (BL62) | 0.997 | | Excellent | 0.996 | Excellent | 0.995 | Excellent |
|  | Taichong (LR3) | 0.999 | | Excellent | 0.994 | Excellent | 0.997 | Excellent |
|  | Xingjian (LR2) | 0.999 | | Excellent | 0.999 | Excellent | 0.996 | Excellent |


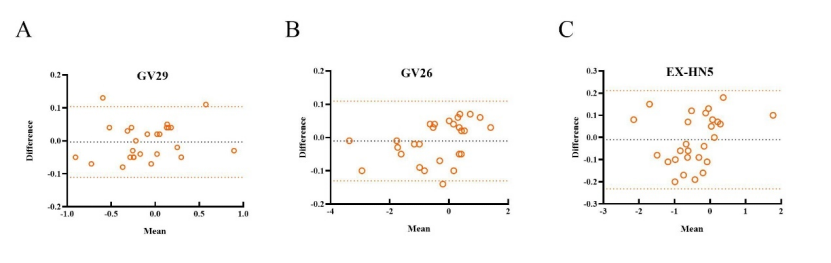


Figure.1 The Bland-Altman plots of the inter-investigator measurements in infrared relative temperature of acupoints on face. **(A)** The Bland-Altman plots of the inter-investigator measurements in Yintang (GV29). **(B)** The Bland-Altman plots of the inter-investigator measurements in Shuigou (GV26). **(C)** The Bland-Altman plots of the inter-investigator measurements in Taiyang (EX-HN5).


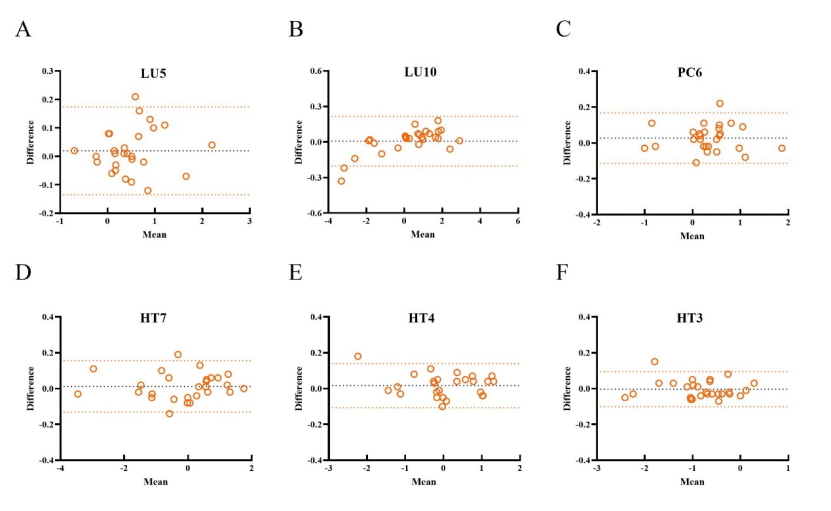


Figure.2 The Bland-Altman plots of the inter-investigator measurements in infrared relative temperature of acupoints on medial upper limb. **(A)** The Bland-Altman plots of the inter-investigator measurements in Chize (LU5). **(B)** The Bland-Altman plots of the inter-investigator measurements in Yuji (LU10). **(C)** The Bland-Altman plots of the inter-investigator measurements in Neiguan (PC6). **(D)** The Bland-Altman plots of the inter-investigator measurements in Shenmen (HT7). **(E)** The Bland-Altman plots of the inter-investigator measurements in Tongli (HT4). **(F)** The Bland-Altman plots of the inter-investigator measurements in Shaohai (HT3).


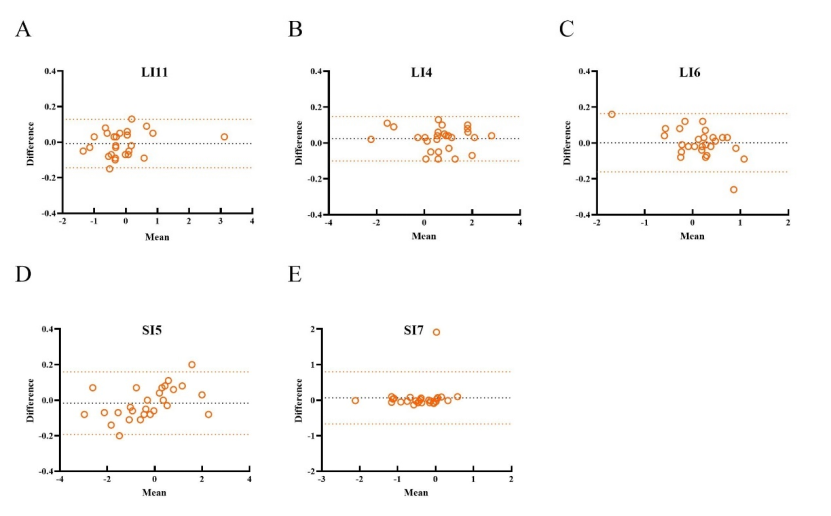


Figure.3 The Bland-Altman plots of the inter-investigator measurements in infrared relative temperature of acupoints on lateral upper limb. **(A)** The Bland-Altman plots of the inter-investigator measurements in Quchi (LI11). **(B)** The Bland-Altman plots of the inter-investigator measurements in Hegu (LI4). **(C)** The Bland-Altman plots of the inter-investigator measurements in Pianli (LI6). **(D)** The Bland-Altman plots of the inter-investigator measurements in Yanggu (SI5). **(E)** The Bland-Altman plots of the inter-investigator measurements in Zhizheng (SI7).


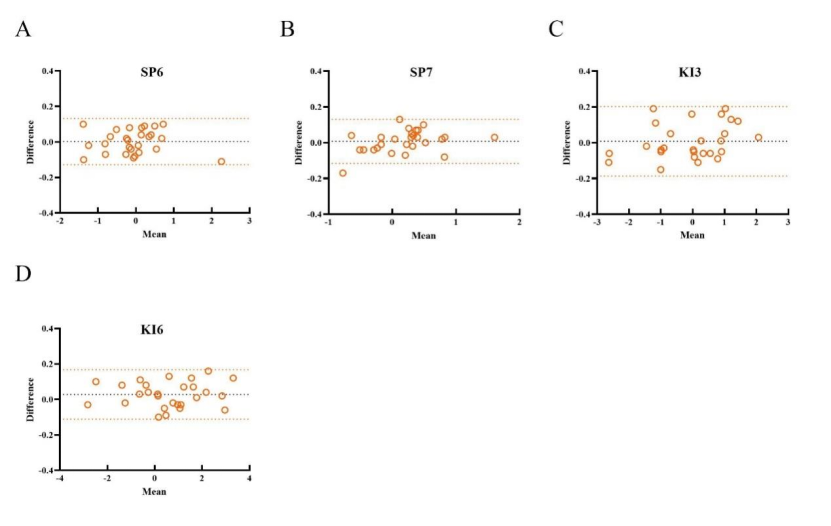


Figure.4 The Bland-Altman plots of the inter-investigator measurements in infrared relative temperature of acupoints on medial lower limb. **(A)** The Bland-Altman plots of the inter-investigator measurements in Sanyinjiao (SP6). **(B)** The Bland-Altman plots of the inter-investigator measurements in Yinlingquan (SP7). **(C)** The Bland-Altman plots of the inter-investigator measurements in Taixi (KI3). **(D)** The Bland-Altman plots of the inter-investigator measurements in Zhaohai (KI6).


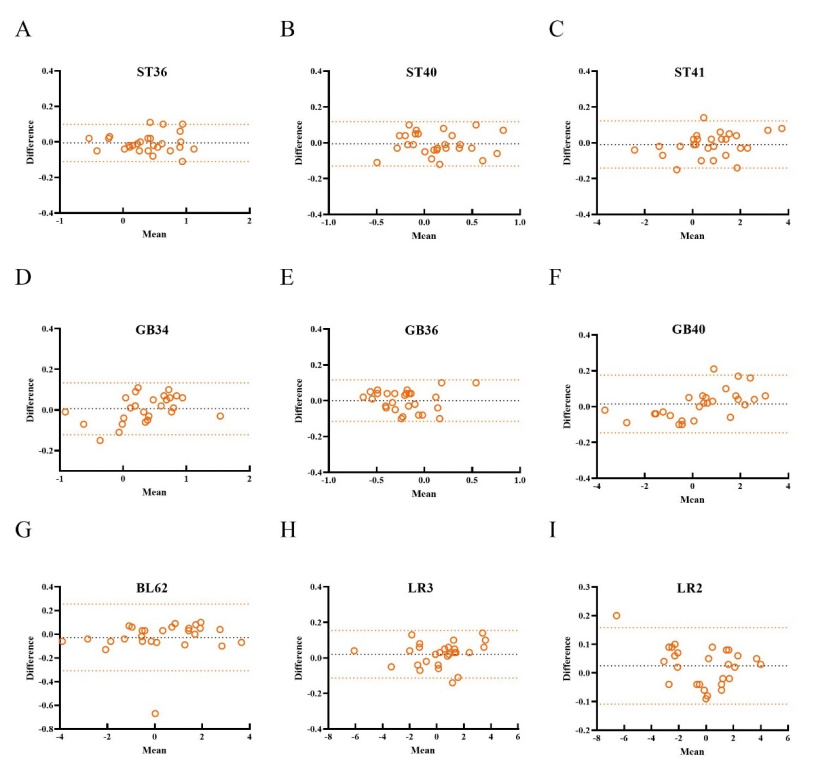


Figure.5 The Bland-Altman plots of the inter-investigator measurements in infrared relative temperature of acupoints on medial lower limb. **(A)** The Bland-Altman plots of the inter-investigator measurements in Zusanli (ST36). **(B)** The Bland-Altman plots of the inter-investigator measurements in Fenglong (ST40). **(C)** The Bland-Altman plots of the inter-investigator measurements in Jiexi (ST41). **(D)** The Bland-Altman plots of the inter-investigator measurements in Yanglingquan (GB34). **(E)** The Bland-Altman plots of the inter-investigator measurements in Waiqiu (GB36). **(F)** The Bland-Altman plots of the inter-investigator measurements in Qiuxu (GB40). **(G)** The Bland-Altman plots of the inter-investigator measurements in Shenmai (BL62). **(H)** The Bland-Altman plots of the inter-investigator measurements in Taichong (LR3). **(I)** The Bland-Altman plots of the inter-investigator measurements in Xingjian (LR2).


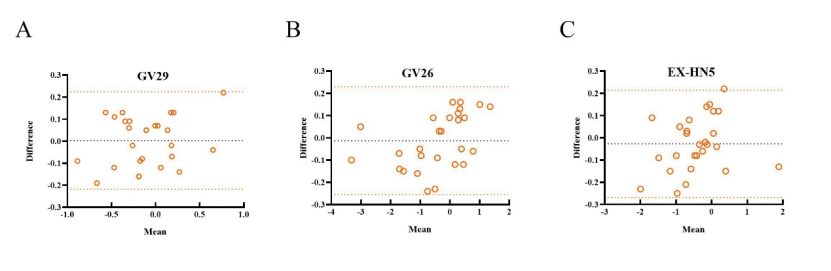


Figure.6 The Bland-Altman plots showing agreement for investigator 1 in infrared relative temperature of acupoints on face. **(A)** The Bland-Altman plots showing agreement for investigator 1 in Yintang (GV29). **(B)** The Bland-Altman plots showing agreement for investigator 1 in Shuigou (GV26). **(C)** The Bland-Altman plots showing agreement for investigator 1 in Taiyang (EX-HN5).


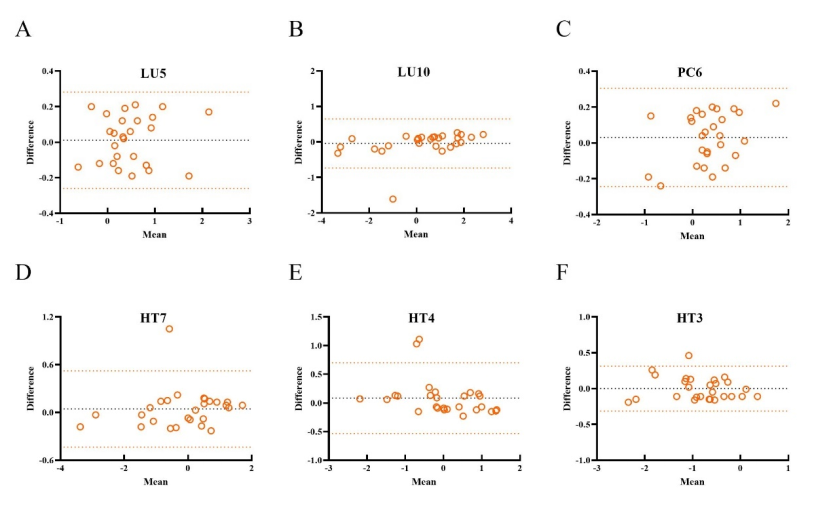


Figure.7 The Bland-Altman plots showing agreement for investigator 1 in infrared relative temperature of acupoints on medial upper limb. **(A)** The Bland-Altman plots showing agreement for investigator 1 in Chize (LU5). **(B)** The Bland-Altman plots showing agreement for investigator 1 in Yuji (LU10). **(C)** The Bland-Altman plots showing agreement for investigator 1 in Neiguan (PC6). **(D)** The Bland-Altman plots showing agreement for investigator 1 in Shenmen (HT7). **(E)** The Bland-Altman plots showing agreement for investigator 1 in Tongli (HT4). **(F)** The Bland-Altman plots showing agreement for investigator 1 in Shaohai (HT3).


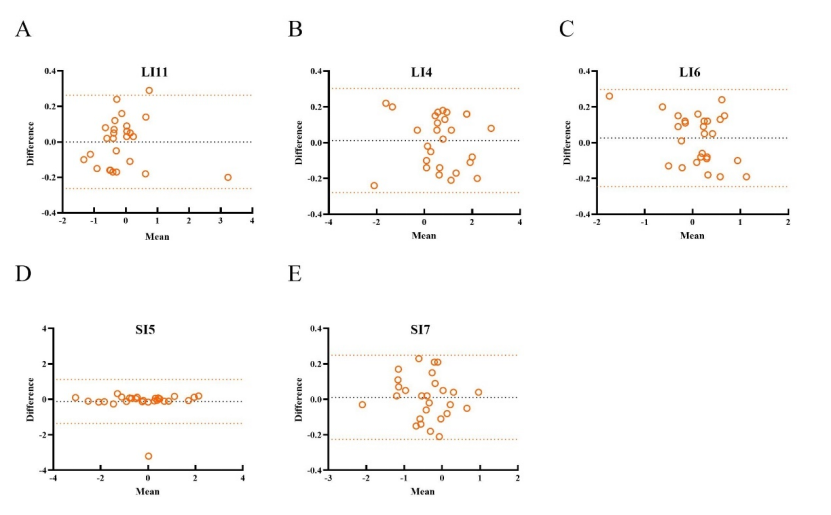


Figure.8 The Bland-Altman plots showing agreement for investigator 1 in infrared relative temperature of acupoints on on lateral upper limb. **(A)** The Bland-Altman plots showing agreement for investigator 1 in Quchi (LI11). **(B)** The Bland-Altman plots showing agreement for investigator 1 in Hegu (LI4). **(C)** The Bland-Altman plots showing agreement for investigator 1 in Pianli (LI6). **(D)** The Bland-Altman plots showing agreement for investigator 1 in Yanggu (SI5). **(E)** The Bland-Altman plots showing agreement for investigator 1 in Zhizheng (SI7).


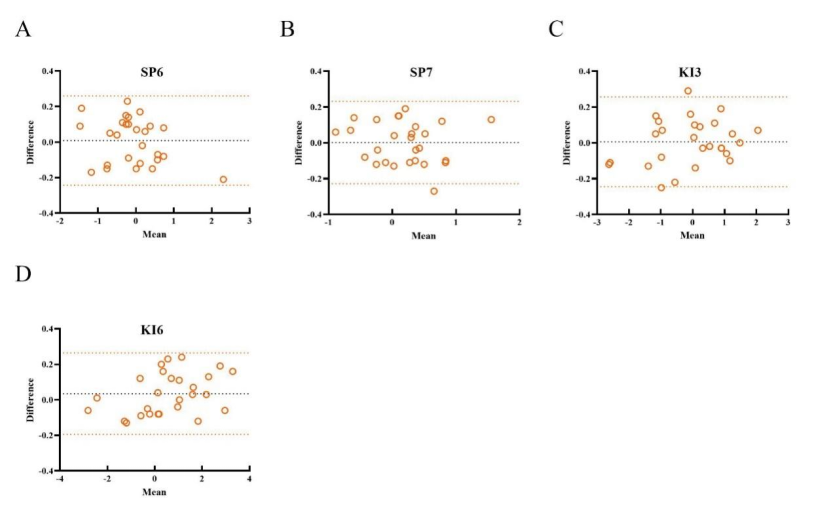


Figure.9 The Bland-Altman plots showing agreement for investigator 1 in infrared relative temperature of acupoints on medial lower limb. **(A)** The Bland-Altman plots showing agreement for investigator 1 in Sanyinjiao (SP6). **(B)** The Bland-Altman plots showing agreement for investigator 1 in Yinlingquan (SP7). **(C)** The Bland-Altman plots showing agreement for investigator 1 in Taixi (KI3). **(D)** The Bland-Altman plots showing agreement for investigator 1 in Zhaohai (KI6).


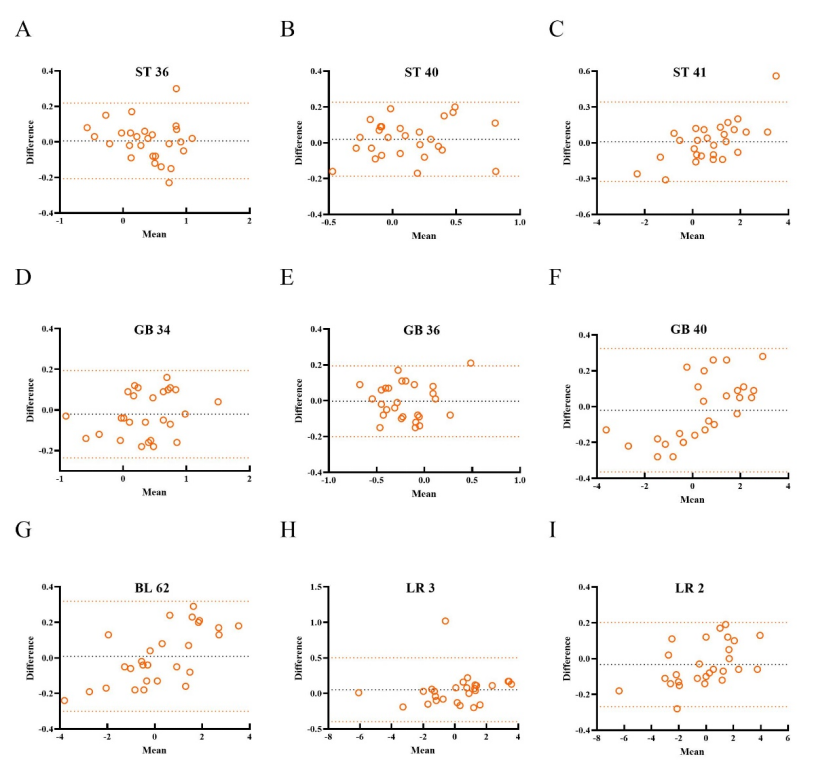


Figure.10 The Bland-Altman plots showing agreement for investigator 1 in infrared relative temperature of acupoints on medial lower limb. **(A)** The Bland-Altman plots showing agreement for investigator 1 in Zusanli (ST36). **(B)** The Bland-Altman plots showing agreement for investigator 1 in Fenglong (ST40). **(C)** The Bland-Altman plots showing agreement for investigator 1 in Jiexi (ST41). **(D)** The Bland-Altman plots showing agreement for investigator 1 in Yanglingquan (GB34). **(E)** The Bland-Altman plots showing agreement for investigator 1 in Waiqiu (GB36). **(F)** The Bland-Altman plots showing agreement for investigator 1 in Qiuxu (GB40). **(G)** The Bland-Altman plots showing agreement for investigator 1 in Shenmai (BL62). **(H)** The Bland-Altman plots showing agreement for investigator 1 in Taichong (LR3). **(I)** The Bland-Altman plots showing agreement for investigator 1 in Xingjian (LR2).


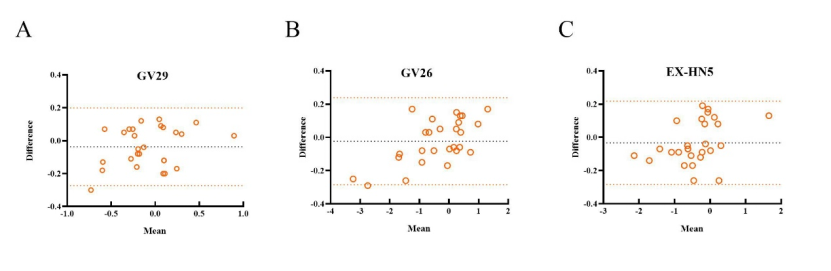


Figure.11 The Bland-Altman plots showing agreement for investigator 2 in infrared relative temperature of acupoints on face. **(A)** The Bland-Altman plots showing agreement for investigator 2 in Yintang (GV29). **(B)** The Bland-Altman plots showing agreement for investigator 2 in Shuigou (GV26). **(C)** The Bland-Altman plots showing agreement for investigator 2 in Taiyang (EX-HN5).


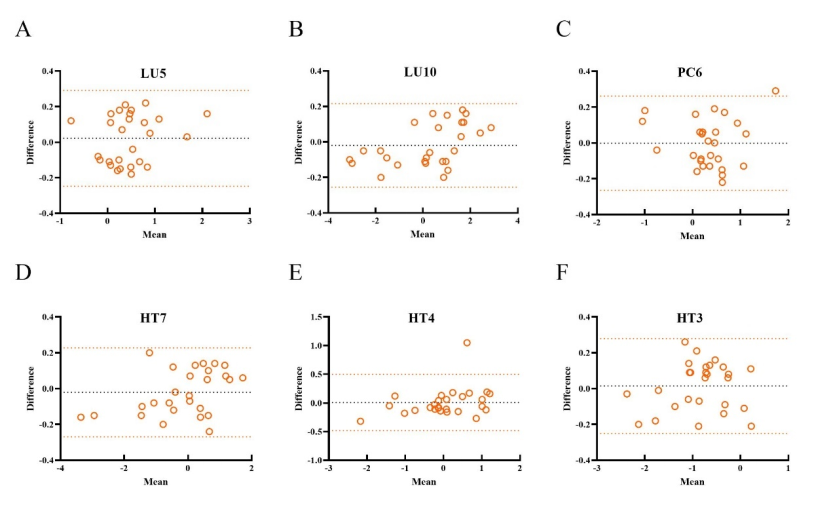


Figure.12 The Bland-Altman plots showing agreement for investigator 2 in infrared relative temperature of acupoints on medial upper limb. **(A)** The Bland-Altman plots showing agreement for investigator 2 in Chize (LU5). **(B)** The Bland-Altman plots showing agreement for investigator 2 in Yuji (LU10). **(C)** The Bland-Altman plots showing agreement for investigator 2 in Neiguan (PC6). **(D)** The Bland-Altman plots showing agreement for investigator 2 in Shenmen (HT7). **(E)** The Bland-Altman plots showing agreement for investigator 2 in Tongli (HT4). **(F)** The Bland-Altman plots showing agreement for investigator 2 in Shaohai (HT3).


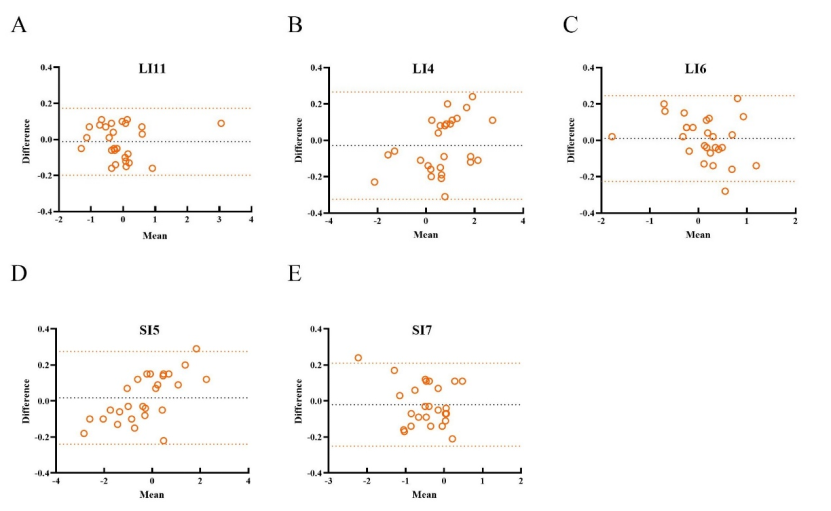


Figure.13 The Bland-Altman plots showing agreement for investigator 2 in infrared relative temperature of acupoints on lateral upper limb. **(A)** The Bland-Altman plots showing agreement for investigator 2 in Quchi (LI11). **(B)** The Bland-Altman plots showing agreement for investigator 2 in Hegu (LI4). **(C)** The Bland-Altman plots showing agreement for investigator 2 in Pianli (LI6). **(D)** The Bland-Altman plots showing agreement for investigator 2 in Yanggu (SI5). **(E)** The Bland-Altman plots showing agreement for investigator 2 in Zhizheng (SI7).


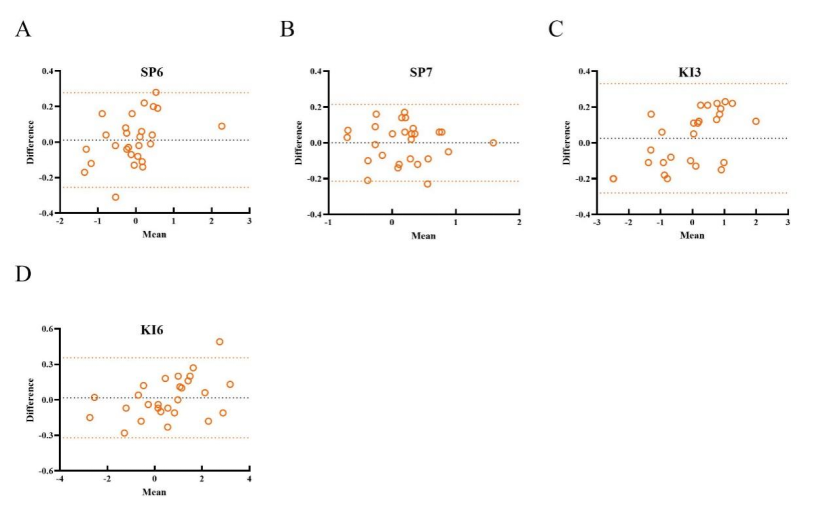


Figure.14 The Bland-Altman plots showing agreement for investigator 2 in infrared relative temperature of acupoints on medial lower limb. **(A)** The Bland-Altman plots showing agreement for investigator 2 in Sanyinjiao (SP6). **(B)** The Bland-Altman plots showing agreement for investigator 2 in Yinlingquan (SP7). **(C)** The Bland-Altman plots showing agreement for investigator 2 in Taixi (KI3). **(D)** The Bland-Altman plots showing agreement for investigator 2 in Zhaohai (KI6).


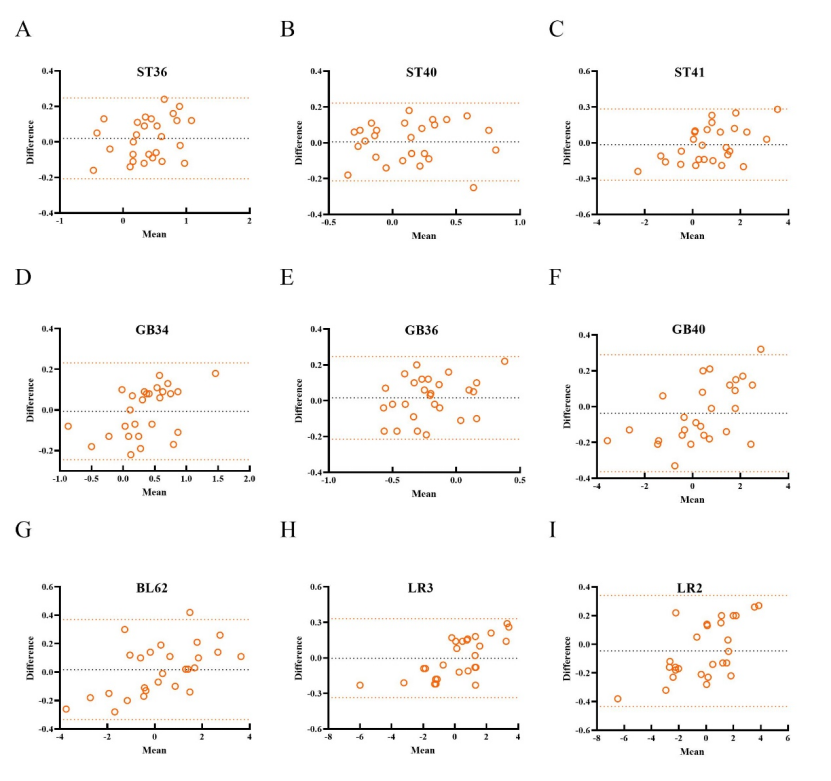


Figure.15 The Bland-Altman plots showing agreement for investigator 2 in infrared relative temperature of acupoints on medial lower limb. **(A)** The Bland-Altman plots showing agreement for investigator 2 in Zusanli (ST36). **(B)** The Bland-Altman plots showing agreement for investigator 2 in Fenglong (ST40). **(C)** The Bland-Altman plots showing agreement for investigator 2 in Jiexi (ST41). **(D)** The Bland-Altman plots showing agreement for investigator 2 in Yanglingquan (GB34). **(E)** The Bland-Altman plots showing agreement for investigator 2 in Waiqiu (GB36). **(F)** The Bland-Altman plots showing agreement for investigator 2 in Qiuxu (GB40). **(G)** The Bland-Altman plots showing agreement for investigator 2 in Shenmai (BL62). **(H)** The Bland-Altman plots showing agreement for investigator 2 in Taichong (LR3). **(I)** The Bland-Altman plots showing agreement for investigator 2 in Xingjian (LR2).
