# Supplementary material for: Acupoint temperature as a biomarker: infrared thermography in the diagnosis of adolescents with major depressive disorder
Source: Front Psychiatry. 2026 Apr 29;17:1806676. doi: 10.3389/fpsyt.2026.1806676 (PMC13168048; doi:10.3389/fpsyt.2026.1806676)
Supplement: Supplementary file 2 [file SupplementaryFile2.docx]

# *Bilateral acupoint temperature symmetry in adolescents with MDD via infrared thermography*

# Materials and methods

## Participants

## Available references suggested that the bilateral same name acupoint temperatures of healthy persons are basically equivalent (1). Therefore, we only detected the bilateral same name acupoint temperatures in adolescents with MDD. From April 2024 to June 2024, adolescents with MDD were recruited from the Third Affiliated Hospital of Zhejiang Chinese Medical University, Hangzhou First People’s Hospital, and Tongde Hospital of Zhejiang Province, and diagnosed based on International Classification of Diseases-10 (ICD-10) by psychiatrists with 20 years of working experience. The Self-rating Depression Scale (SDS) was completed by all subjects to assess the severity of depressive disorder. Only patients with SDS score greater than 53 were included in the study. The exclusion criteria included: (1) Severe anxiety, schizophrenia, or other serious mental illnesses; (2) There are pigmentation, redness, infection or scarring on the skin at the site of detection. (3) Participants with severe systemic diseases and their complications, serious infections, and other major medical conditions; (4) Participants who were pregnant, lactating, during, or in proximity (± 2 days) to their menstruation or ovulation; (5) Participants with a body temperature ≥ 37.3°C; (6) Participants who did not complete the IRT measurement. All subjects volunteered to participate in this study provided assent, while parents or guardians provided informed consent.

## Sample size estimation was performed a priori using G*Power software (version 3.1.9.7) for paired t-test analysis. The calculation was conducted with a two-tailed α level of 0.05 and 80% statistical power. The analysis determined that 31 paired samples would be required to detect significant differences. To account for potential data attrition during experimental procedures, the final sample size was increased to 33 pairs.

## Acquisition of IRT images

IRT image acquisition was performed following the standardized protocol detailed in Materials and methods.

## The detection acupoints and analytical method

Only Taiyang (EX-HN5), Yanggu (SI5), Waiqiu (GB36) which were involved in both models were selected for the bilateral same name acupoint temperature detection.

The analytical methodology strictly adhered to the experimental procedures delineated in Materials and methods.

## Statistical Analysis

SPSS 25.0 (IBM SPSS Statistics for Windows, USA) was used for statistical analysis. All normally distributed data are presented as Mean ± SD, and non-normally distributed data are presented as M (Q₁, Q₃). Normally distributed data (assessed via Shapiro-Wilk test) were analyzed using paired t-tests, while non-parametric Wilcoxon rank-sum tests were applied to skewed distributions. Significance was set at *P*<0.05.

# Result

T Initially, 33 adolescents with MDD were enrolled. Among them, 3 adolescents failed to persist through the examination, 30 subjects were finally included in the study. There were no significant differences in the bilateral same name acupoint temperatures for adolescents with MDD, as illustrates in Table 1.

**References**

1. Wei HN, Jiang LP, Xiong B, Zhou S, Yu L, Huang YM, et al. Characteristic patterns of normal meridian acupoint temperature. *J Chin Med Assoc* (2017) 80:419-426. doi: 10.1016/j.jcma.2016.12.007

Table 1 Infrared relative temperature of bilateral same name acupoint for adolescents with MDD [Mean ± SD, M (Q1, Q3)]

| Acupoints | left side (n=30) | right side (n=30) | *t*/*z* | *P* value |
| --- | --- | --- | --- | --- |
| Taiyang (EX-HN5) | -0.22 (-0.58, 0.31) | -0.22 (-0.53, 0.26) | -1.73 | 0.084 |
| Yanggu (SI5) | -0.17 ± 1.18 | -0.16 ± 1.11 | -0.50 | 0.621 |
| Waiqiu (GB36) | -0.07 (-0.18, 0.21) | 0.01 (-0.19, 0.17) | -1.14 | 0.253 |
